# Specialty-associated differences in intensive care unit rotation exit examination performance: a single-center retrospective cross-sectional study

**DOI:** 10.3389/fmed.2026.1960270

**Published:** 2026-10-07

**Authors:** Yanche Lu, Ruijie Lin, Qian Yu, Qiuyu Zheng, Lianmin Ye

**Affiliations:** 1 Wenzhou Medical University, Wenzhou, China; 2 The Second Affiliated Hospital and Yuying Children's Hospital of Wenzhou Medical University, Wenzhou, China; 3 Wenzhou Medical University Renji College, Wenzhou, China; 4 The First Affiliated Hospital of Wenzhou Medical University, Wenzhou, China

**Keywords:** confounding, educational measurement, graduate medical education, intensive care unit rotation, specialty differences, training needs assessment

## Abstract

**Background:**

Intensive care unit (ICU) rotations bring together residents from multiple specialties. Comparisons of exit performance may be distorted when departments differ in postgraduate-year (PGY) composition and when non-equivalent examination forms are used across PGY levels.

**Methods:**

We conducted a retrospective cross-sectional analysis of ICU exit examination records from 412 residents at a tertiary teaching hospital in China between November 2025 and April 2026. Three 100-item, 60-min examinations containing four question types were administered according to PGY. Departmental differences were evaluated using four complementary strategies: a same-examination subgroup of 227 PGY-2 residents, within-examination standardization, analysis of covariance adjusting for the combined PGY/form grouping, and hierarchical regression. Item characteristics, version-specific reliability, and exploratory differential item functioning were assessed retrospectively.

**Results:**

In the same-examination subgroup, the total-score effect size was *η*^2^ = 0.098 (*p* < 0.001), with surgery having the lowest mean score (75.9; SD 9.1; *n* = 77; 95% CI 73.9–78.0). Department explained an additional 7.6% of total-score variance after adjustment for PGY/form. The descriptive attenuation ratio was 21.3% for total scores, ranging from 7.3% for case-series items to 80.2% for basic-knowledge items. These ratios do not identify separate training-year or examination-version effects. The largest negative case-analysis gaps occurred in surgery and anesthesiology.

**Conclusion:**

Specialty-associated differences in examination performance were observed within this single-center educational setting and using this assessment instrument. The absence of baseline scores and incomplete evidence of cross-form comparability preclude causal or inherent-specialty interpretations; prospective baseline-controlled studies are needed before specialty-tailored interventions are adopted.

## Introduction

China's standardized residency training framework emphasizes the development and assessment of core clinical competencies (1–3). In the participating hospital, residents from multiple specialties completed a common intensive care unit (ICU) rotation. Whether this rotation provides comparable learning opportunities across trainees with different prior clinical exposure warrants examination.

Prior clinical exposure may differ across trainees, but specialty membership alone does not establish how closely their previous experience matches ICU practice. The distance between a learning context and a trainee's prior practice has been described as learning-transfer distance and may influence how readily knowledge is applied in a new setting (4, 5).

Evidence on cross-specialty ICU rotation outcomes remains limited. Piquette et al. assessed resident competencies before and after short ICU rotations but did not stratify outcomes by home specialty (6). A nationwide Japanese study subsequently showed that residents pursuing different specialties differed in general clinical proficiency (7). More recent work has emphasized multimodal and longitudinal assessment in critical care education (8), the perceived value of specialty-tailored short ICU rotations (9), the distinct information yielded by different in-training examination formats (10), and course-specific variation in theoretical examination performance (11). Together, these studies support closer attention to both trainee background and assessment design.

Score interpretation also depends on the validity of the comparison being made. Validity is not an intrinsic property of a test; it is an argument about the interpretation and use of scores that must be supported by appropriate evidence (12). Longitudinal progress-test research illustrates the value of modeling training-related change rather than relying on a single cross-sectional score (13). When different forms are administered, comparability cannot be assumed solely because the forms cover the same content; explicit linkage or equating is ordinarily needed to support interchangeable score interpretations (14).

In this dataset, examination form and PGY were linked by design: each PGY completed a different form. The proportion of case-series items increased from 29% in Exam 1 to41% in Exam 3, and PGY composition differed across specialties. We therefore made the same-form PGY-2 comparison the primary analysis and used pooled, standardized, and adjusted analyses as complementary descriptions. Our aim was to describe observed differences in examination performance and their sensitivity to the analytical approach, rather than to isolate independent effects of examination version and training year or infer specialty-specific learning gains.

## Materials and methods

### Study design and participants

This retrospective cross-sectional study used ICU rotation exit examination records from the First Affiliated Hospital of Wenzhou Medical University, a tertiary academic medical center in Zhejiang, China. The examinations were administered to residents who had completed a 1-month ICU rotation. Six consecutive monthly rotation batches were examined from November 29, 2025, to April 29, 2026. The six regular examination dates were November 29 and December 27, 2025, and January 29, February 27, March 27, and April 29, 2026. Individual resits were arranged separately. The dataset contained 437 records from 412 residents. Twenty-three residents contributed two or three records, accounting for 25 additional records. For each resident, we retained the first record in the established dataset; this was also the lowest-PGY record in every case. The primary dataset therefore contained one record per resident (*n* = 412), while all 437 records were retained for a complementary record-level analysis. Reporting follows STROBE (15; Supplementary Table S1).

Available dated administration records span November 29, 2025, to April 29, 2026, for each of Exams 1–3. The register contains Exam 2 and Exam 3 records on all six regular dates, and Exam 1 records on November 29 and December 27, 2025, and January 29, February 27, and April 29, 2026. The absence of a March Exam 1 entry in this incomplete register does not establish that the form was not administered that month. Version-by-date coverage is shown in Supplementary Table S6; these administrative counts are not the denominators of the 412-resident or 437-record analyses.

The Ethics Committee in Clinical Research of the First Affiliated Hospital of Wenzhou Medical University approved the study on August 7, 2026 (approval no. KY2026-R287). The committee waived the requirement for informed consent because the study used fully anonymized, routinely collected examination records and involved no clinical intervention or identifiable personal data.

### Design limitation

Only post-rotation examination scores were available. Observed specialty-associated differences may therefore reflect pre-existing knowledge, previous exposure, learning during the rotation, or unmeasured contextual factors. Restricting the primary comparison to Exam 2 holds the administered form and PGY constant within that subgroup. In pooled analyses, PGY adjustment addresses the combined PGY/form grouping; because assignment was deterministic, this dataset cannot separate their independent effects. None of these approaches establishes a causal effect of rotation training.

### Examination instruments

The three versions, locally named ‘ICU Rotation Exit Examination 1,’ ‘ICU Rotation Exit Examination 2,’ and ‘ICU Rotation Exit Examination 3’ (Exams 1, 2, and 3), were assigned to PGY-1, PGY-2, and PGY-3 residents, respectively. Each examination contained 100 items, had a 60-min time limit, and awarded 1 point per correct item, giving a maximum total of 100. The A1/A2/A3–A4/case-analysis item counts were 30/30/29/11 for Exam 1, 20/30/31/19 for Exam 2, and 20/20/41/19 for Exam 3. These percentages describe item-format weighting, not independently verified content-domain weighting. A1 and A2 items used single-choice formats, A3/A4 items used case-series single-choice formats, and case-analysis items required selection of the complete set of correct options. Case-analysis questions occupied Q90–Q100 in Exam 1 and Q82–Q100 in Exams 2 and 3. Their response-option counts ranged from 6 to 9, 5 to 9, and 5 to 9, respectively (Supplementary Table S6). Under the author-confirmed all-or-none scoring rule, an exactly correct response received 1 point; incomplete or incorrect responses received 0, without partial credit.

### Examination development and comparability

No pilot testing, systematic test-blueprint development, or formal review of content-domain weights was undertaken before administration. A documented blueprint-development process, approved content-domain weights, and cognitive-level mapping therefore cannot be reconstructed from the available examinations. Formal score equating and psychometric equivalence across the three versions have not been established. Equal item counts and time limits do not establish equivalent difficulty, content representation, or cognitive demands. The item-format proportions reported in Supplementary Table S6 are not content-domain weights. Item difficulty, item-rest discrimination, and version-specific reliability were examined retrospectively for this revision; these analyses should not be interpreted as pre-administration validation. Neither within-form z standardization nor inclusion of PGY/form indicators constitutes test equating.

### Scored-item analyses and reliability

The latest matrices contained 437 records with 100 binary scored items each and no missing cells. Identity/form mapping and record-retention indices were checked against the locked datasets. Item sums agreed with every authoritative total score; component sums agreed with rounded component percentages. Item difficulty was defined as the observed proportion correct, *p*_i_. Discrimination was the correlation between the item score and the sum of the other 99 items (corrected item-total or item-rest correlation). Constant items or zero-variance rest scores were not assigned a correlation. The system-level difficulty coefficients were not used as item *p*_i_ values. Only 0/1 scored outcomes were available; actual selected options were not exported, so distractor-functioning analysis could not be performed.

Cronbach's alpha, equivalent to KR-20 for these complete binary items, was calculated separately for each full 100-item form and for its case-analysis subscale (16, 17). Form-record estimates used *n* = 27, 235, and 175; estimates from the deduplicated cohort used *n* = 27, 227, and 158. Percentile 95% confidence intervals used 2,000 bootstrap samples (seed 20260907, restarted within each form/cohort), resampling residents as clusters so that within-form repeat records were retained together. No items were deleted to increase reliability. Alpha assesses internal consistency and does not establish unidimensionality, content validity, or cross-form equivalence.

### Exploratory differential item functioning

DIF screening was restricted to the five Exam 2 specialties with *n* ≥ 20 (*n* = 221), including orthopedics; critical care medicine (*n* = 6) was retained in group descriptions but excluded from this unstable item-level comparison. For each item, a logistic model containing the standardized rest score was compared with models adding specialty and specialty-by-rest-score interactions. The primary omnibus likelihood-ratio test compared the rest-score model with the full interaction model. Uniform and nonuniform components were examined separately. A minimum of five correct and five incorrect responses per specialty was required; matching-score overlap, convergence, design rank, extreme coefficients/standard errors, and fitted-probability or separation warnings were also checked. Items failing these criteria were reported as not stably estimable.

Benjamini–Hochberg correction used a family of 100 items for each of the omnibus, uniform, and nonuniform screens. Non-estimable tests were represented internally by *p* = 1 to preserve the family size but displayed as not estimable; q < 0.05 defined a statistical flag. *Δ*Nagelkerke R^2^ quantified the omnibus model increment. The rest score was a provisional matching variable, not a validated anchor score. These exploratory analyses were undertaken in response to peer review and should not be described as prospectively preregistered.

### Department grouping

The original analytical coding grouped residents into six categories: internal medicine (*n* = 156; general internal medicine, endocrinology, and medical oncology), surgery (*n* = 98; general surgery and surgical subspecialties), anesthesiology (*n* = 31), orthopedics (*n* = 29), critical care medicine (*n* = 20), and other clinical specialties (*n* = 78). The other clinical group comprised 11 recorded categories not classified within the five core groups (individual *n* = 1–25; Supplementary Table S2) and was combined because most category-specific samples were too small for separate analysis. These analytical categories are not a universal specialty taxonomy. Four residents recorded as surgery with a plastic-surgery direction were already included in Surgery; one separately recorded as plastic surgery was coded as Other Clinical. Original coding was retained as the reference, with alternative classifications reported in Supplementary Table S10.

### Analytical strategy

The primary analysis compared total scores among the six specialties in the Exam 2/PGY-2 subgroup (*n* = 227) using one-way ANOVA and Tukey HSD pairwise comparisons. We report mean, sample SD, n, and 95% t confidence intervals, calculated from unrounded data. The median-centered Levene/Brown–Forsythe test assessed variance heterogeneity. A further same-form analysis retained all five specialties with *n* ≥ 20 (*n* = 221), including orthopedics (*n* = 21), and excluded only critical care medicine (*n* = 6).

Complementary analyses standardized total scores within each examination form and fitted an additive model with specialty and categorical PGY indicators. The specialty-by-PGY interaction was evaluated in a nested model. Hierarchical regression first entered PGY indicators and then specialty indicators. Because PGY identified the form, these models describe the combined PGY/form structure rather than independent effects. We retained the existing attenuation ratio as a descriptive comparison of crude *η*^2^ and adjusted *Δ*R^2^, not a causal attribution. The 437-record analysis was retained only as a non-independent record-level reference. All-record estimates do not constitute an independent replication. A further descriptive check retained the last record for each resident in the established dataset and compared group sizes, total-score means, and the overall ANOVA with first-record retention (Supplementary Table S11). First and last denote dataset order, not independently verified chronological examination dates. Classification sensitivity analyses merged orthopedics and the separately coded plastic-surgery resident into Surgery, then additionally reassigned neurology and infectious diseases to Internal Medicine. A further source-informed scenario reassigned one PGY-3 record with a non-specialty status label to Anesthesiology. Individual scores were unchanged; original and alternative classifications are distinguished in Supplementary Table S10.

### Gap matrix

In the PGY-2 subgroup, each cell of the 6 × 4 descriptive gap matrix was the specialty mean minus the overall PGY-2 mean for that item format, in percentage points. The overall mean includes the specialty being described and is not an independent control group. The previously used labels—critical (< −8), high (−8 to < −5), moderate (−5 to < −2), and low (−2 to < 0)—were retained solely as heuristic display categories. They are not validated diagnostic thresholds or evidence of curricular deficits. No display category was assigned to critical care because only six residents contributed. The 24 cells were exploratory descriptions without multiplicity-adjusted inference.

### Statistical software and use of generative AI

Revision analyses used R 4.6.0 and Python with NumPy and pandas; scripts, input hashes, retained-record indices, and numerical outputs were archived. Tests were two-sided, with *α* = 0.05. ANOVA effect sizes are *η*^2^, regression contributions are *Δ*R^2^. These estimands are not interchangeable. Conventional *η*^2^ benchmarks were used only descriptively (18). Tukey HSD controlled the family-wise error rate for the 15 primary pairwise comparisons. DIF false-discovery-rate procedures are described separately above. Mean CIs use the t distribution; alpha CIs use the bootstrap procedure described above. Sparse *n* = 1 subgroups have no estimable SD or mean CI. For the additive models, adjusted means were standardized to the observed PGY distribution of the 412-resident cohort (27/412, 227/412, and 158/412 for PGY-1–3). Model-based 95% t intervals and regression coefficients are reported in Supplementary Table S5. Raw group SDs describe observed scores; model-adjusted estimates are accompanied by SEs, not relabeled raw SDs.

During original manuscript preparation, the authors used Anthropic Claude (Claude Opus 4.6; Anthropic PBC, San Francisco, CA, USA; https://www.anthropic.com/claude) and OpenAI Codex (GPT-5.6 Sol; OpenAI, San Francisco, CA, USA; https://openai.com/codex/) for language editing, formatting, and preparation of figures and tables from the authors’ data and prespecified analyses. During revision, OpenAI Codex (GPT-6; OpenAI) also assisted with analysis-code preparation and reviewer-response drafting. The authors checked all outputs and take responsibility for the content.

## Results

### Sample composition

The 412 unique residents comprised 156 from internal medicine (37.9%), 98 from surgery (23.8%), 78 from other clinical specialties (18.9%), 31 from anesthesiology (7.5%), 29 from orthopedics (7.0%), and 20 from critical care medicine (4.9%). PGY composition differed substantially across departments: 64.7% of internal medicine residents were PGY-3, 78.6% of surgery residents were PGY-2, and 55.0% of critical care residents were PGY-1 (Table 1). The PGY-2 same-examination subgroup included 227 residents: surgery, 77; internal medicine, 54; other clinical specialties, 49; orthopedics, 21; anesthesiology, 20; and critical care medicine, 6.

**Table 1:** Demographic characteristics of resident physicians by department (*n* = 412).

Department	*n* (%)	PGY-1	PGY-2	PGY-3	Total score, mean ± SD	95% CI
Internal medicine	156 (37.9)	1	54	101	83.0 ± 8.0	81.7–84.2
Surgery	98 (23.8)	2	77	19	76.9 ± 8.8	75.1–78.6
Anesthesiology	31 (7.5)	1	20	10	77.2 ± 8.0	74.3–80.2
Orthopedics	29 (7.0)	2	21	6	82.3 ± 8.2	79.2–85.4
Critical care	20 (4.9)	11	6	3	80.6 ± 8.3	76.7–84.4
Other clinical	78 (18.9)	10	49	19	82.3 ± 7.9	80.5–84.1

PGY, postgraduate year; SD, standard deviation; CI, confidence interval.

### Observed specialty-associated differences in total scores

In the primary same-form analysis, total scores differed across the six specialties, F(5,221) = 4.81, *η*^2^ = 0.098, *p* = 0.00034. Surgery had the lowest mean (75.9, SD 9.1; *n* = 77; 95% CI 73.9–78.0). Tukey-adjusted contrasts favored internal medicine over surgery by 6.1 points (95% CI 1.6–10.6; *p* = 0.0018), orthopedics over surgery by 7.2 points (95% CI 0.9–13.4; *p* = 0.0145), and other clinical specialties over surgery by 5.6 points (95% CI 0.9–10.2; *p* = 0.0085). Figure 1 shows the total-score comparisons; Figures 2 and 3 present the descriptive score gaps and score distributions with variance decomposition, respectively. All specialty descriptives and all 15 pairwise contrasts are provided in Supplementary Table S5. The median-centered Levene/Brown–Forsythe test gave F(5,221) = 0.94, *p* = 0.455. The five-specialty analysis retaining orthopedics gave F(4,216) = 6.03, *η*^2^ = 0.100, *p* = 0.00013 (Supplementary Table S3).

**Figure 1:**
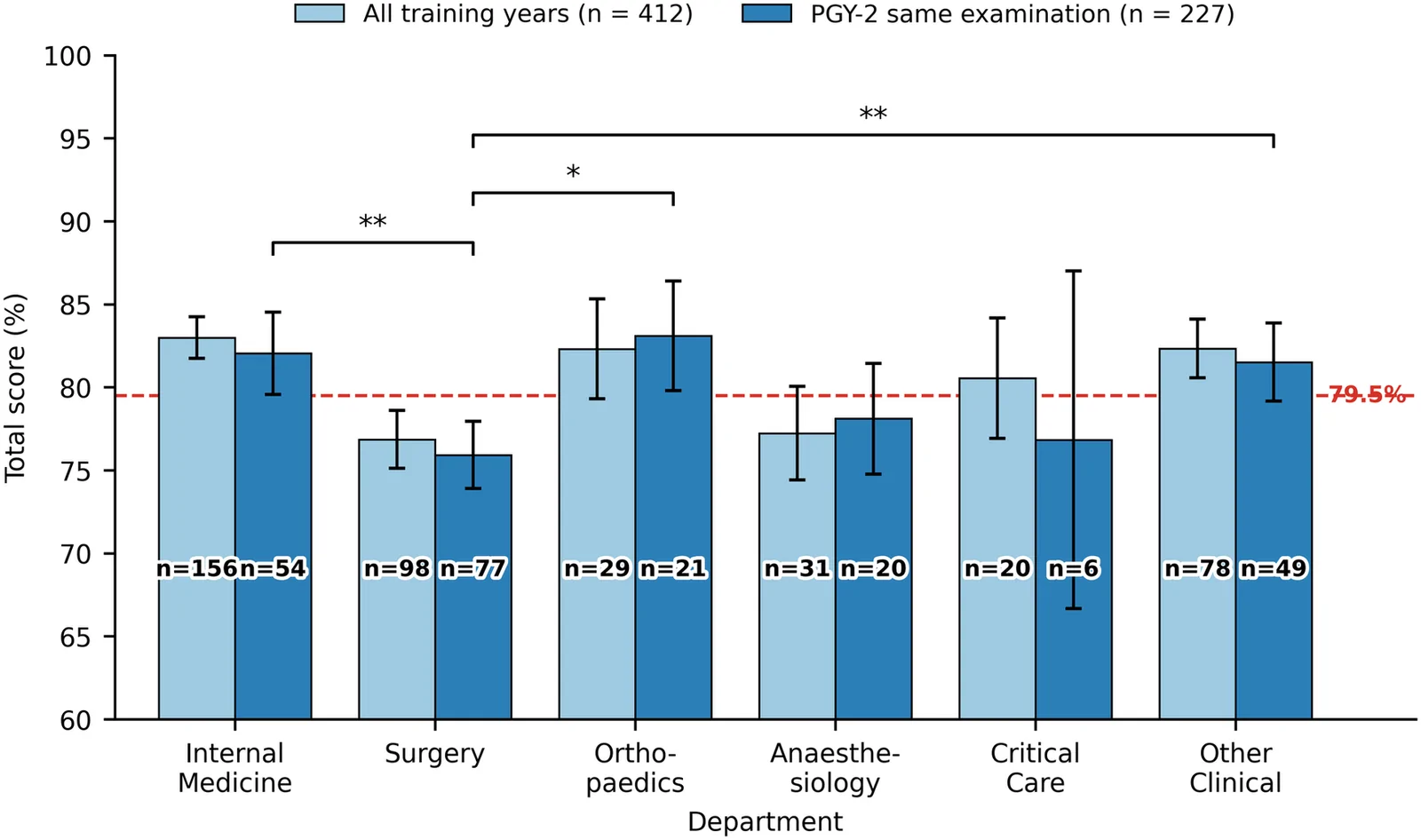
Department performance in the crude and same-examination comparisons. Light bars represent all training years pooled (*n* = 412); dark bars represent the PGY-2 same-examination subgroup (*n* = 227). Error bars show 95% confidence intervals, and the dashed line marks the PGY-2 grand mean (79.5%). *p < 0.05; **p < 0.01 (Tukey honestly significant difference test).

**Figure 2:**
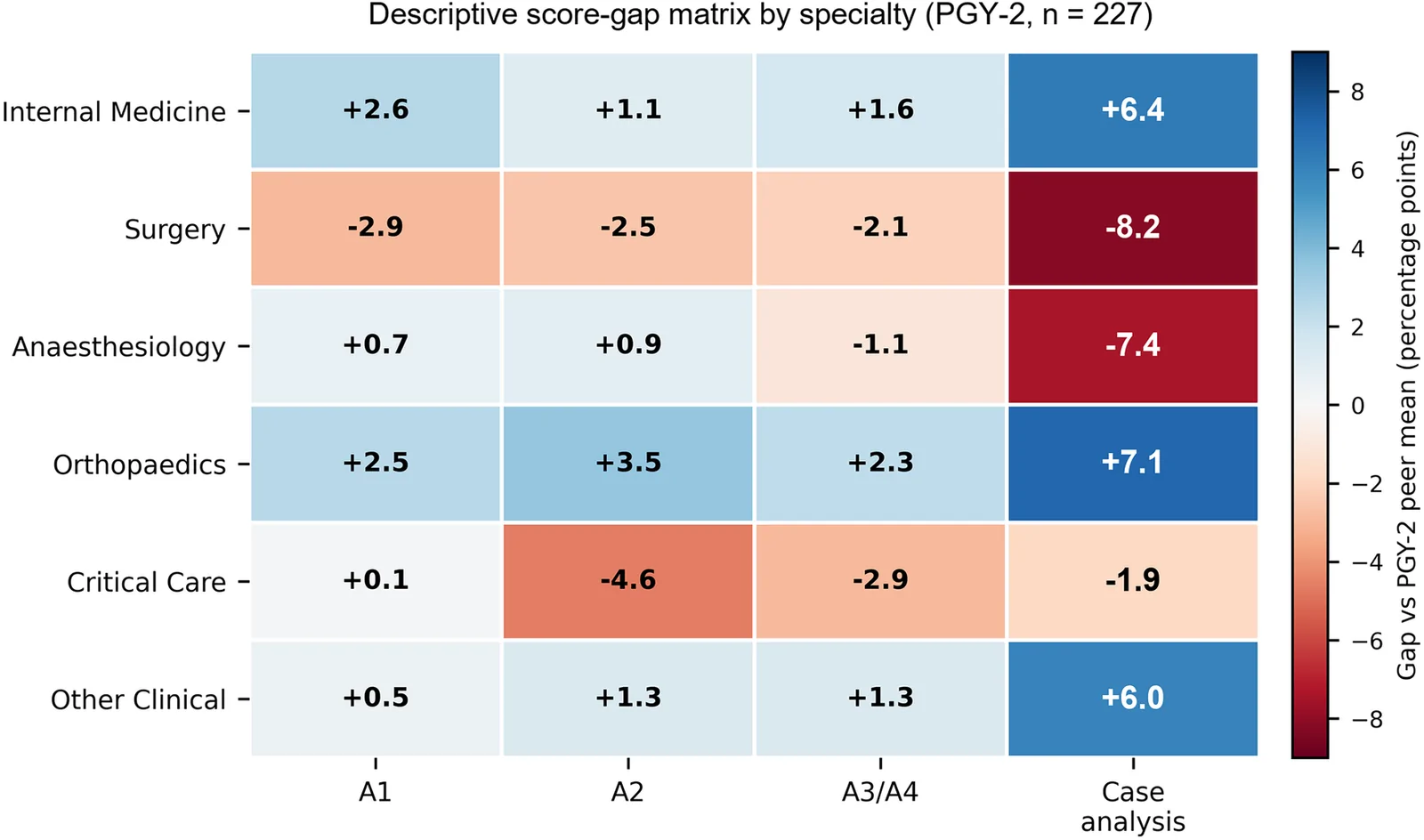
Exploratory score-gap heatmap by specialty and item format in the PGY-2 same-form subgroup (*n* = 227). Each cell is the specialty mean minus the overall PGY-2 mean, in percentage points. Case-analysis values incorporate the three source-verified component corrections. Negative gaps are descriptive findings, not validated training-deficit classifications.

**Figure 3:**
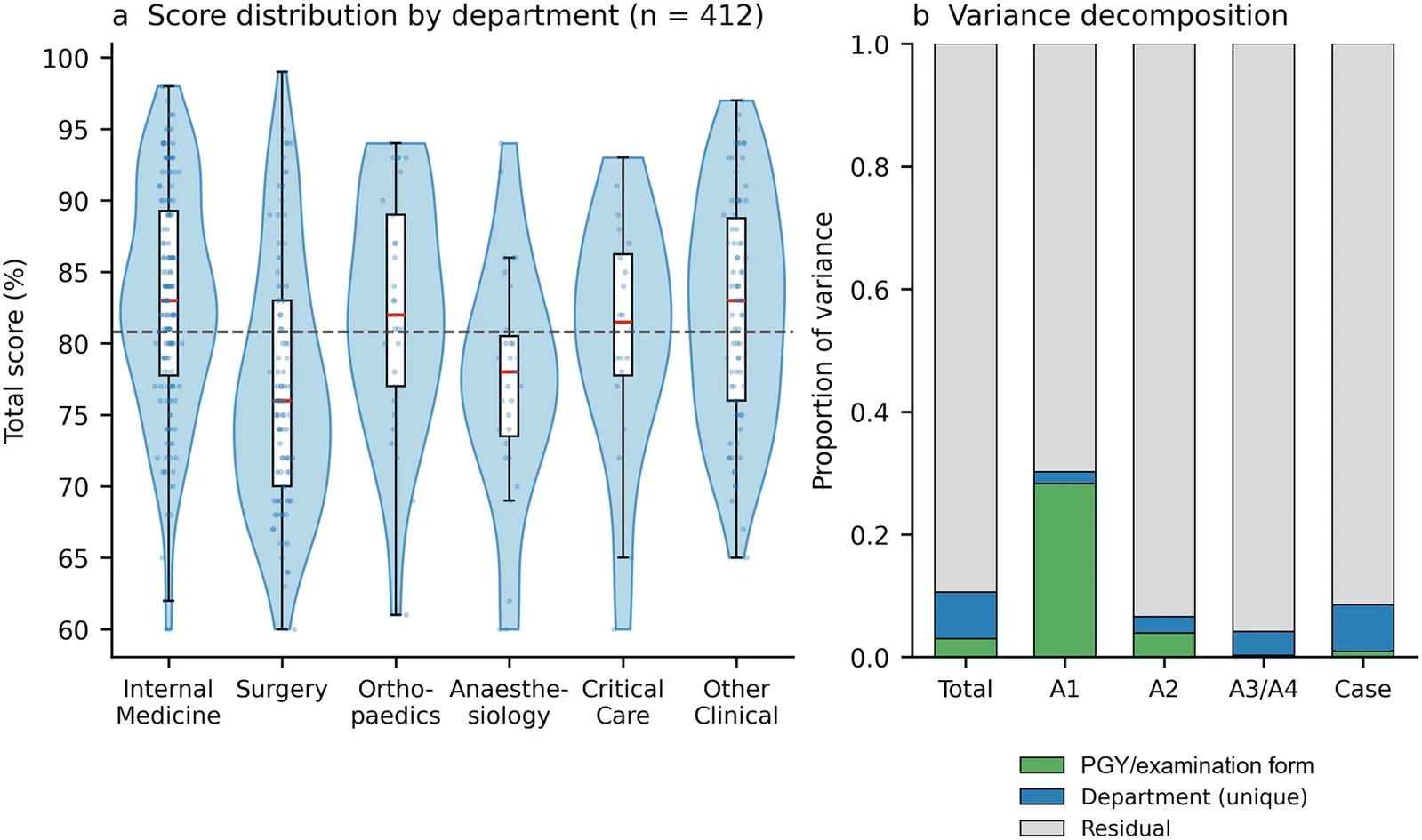
Score distributions and variance decomposition. **(a)** Individual total scores by specialty (*n* = 412). **(b)** Variance associated with the combined PGY/form grouping, the additional specialty contribution after adjustment, and residual variance for each score. Case-analysis decomposition uses the corrected component data.

### Variance partitioning

In the 412-resident dataset, the combined PGY/form grouping explained 3.0% of total-score variance. Adding specialty increased R^2^ to 10.6%, giving *Δ*R^2^ = 0.076 (Table 2; Figure 3B). Relative to crude *η*^2^ = 0.096, this corresponded to a descriptive attenuation ratio of 21.3%. Because PGY and form were inseparable and the models do not identify causal effects, this ratio is not an estimate of the proportion causally attributable to training year. The corresponding ratios were 80.2% for A1 and 7.3% for A3/A4 scores. For case-analysis scores, the adjusted contribution exceeded the crude association, consistent with statistical suppression rather than positive attenuation.

**Table 2:** Variance decomposition of the department effect by question type (hierarchical regression, *n* = 412).

Outcome	R^2^ (PGY/form)	R^2^ (full model)	*Δ*R^2^ department	*η*^2^ crude	Descriptive attenuation (%)
Total score	0.030	0.106	0.076	0.096	21.3
A1 basic knowledge	0.283	0.302	0.019	0.097	80.2
A2 case-based	0.039	0.066	0.027	0.046	41.9
A3/A4 case-series	0.003	0.042	0.039	0.042	7.3
Case analysis	0.008	0.085	0.076	0.062	—^a^

PGY, postgraduate year. The PGY indicators also identify examination form because assignment was deterministic. *Δ*R^2^ is the increase in R^2^ on adding specialty. The descriptive attenuation ratio is (crude η^2^−*Δ*R^2^)/crude η^2^; it is not a causal confounding fraction.

a For case analysis, *Δ*R^2^ exceeds crude η^2^ (suppression), so a positive attenuation percentage is not reported.

Across the four strategies displayed in Supplementary Table S4, the six pairwise Spearman correlations between specialty mean-score rankings ranged from 0.714 to 1.000. Within-form standardization gave F(5,406) = 6.17, *η*^2^ = 0.071; the 437-record reference gave F(5,431) = 7.70, *η*^2^ = 0.082. The specialty-by-PGY interaction was F(10,394) = 0.79, *p* = 0.638. Anesthesiology, rather than surgery, had the lowest within-form standardized mean (−0.42 versus −0.36). The 25 additional records comprised 15 coded as anesthesiology, seven as critical care, and three as internal medicine; group means before and after deduplication are reported in Supplementary Table S11. Additional records represented 32.6% (15/46) of anesthesiology records and 25.9% (7/27) of critical care records; these denominators are records, not residents. Last-record retention gave F(5,406) = 7.06, *η*^2^ = 0.080, *p* < 0.001, with unchanged raw-mean ranking and a maximum first-to-last group-mean change of 0.40 points (Supplementary Table S11). Agreement across these descriptions does not validate cross-form comparability or identify the source of the association.

With orthopedics and plastic surgery merged into Surgery, the same-form association was smaller, F(4,222) = 3.17, *p* = 0.0148, *η*^2^ = 0.054. The merged Surgery mean was 77.46 (SD 9.23; *n* = 98; 95% CI 75.61–79.31), compared with 75.92 under the original coding; critical care had the lowest mean in this alternative grouping. Additional classification checks are reported in Supplementary Table S10.

### Associations by item format

In the PGY-2 subgroup, the case-analysis association was F(5,221) = 4.54, *η*^2^ = 0.093, *p* = 0.00058. The corresponding *η*^2^ values were 0.043 for A1, 0.048 for A2, and 0.048 for A3/A4 scores. These are between-specialty associations at the format-score level, not item-discrimination coefficients. The full-sample crude case-analysis *η*^2^ was 0.062 because it used a different cohort and model (Table 2). Three previously incorrect case-component entries were reconciled to 9 correct case items each; their authoritative total scores remained 92, 84, and 72. The corrected component scores, without changing totals, underlie the revised case-analysis results.

### Item characteristics and internal consistency

Full-form alpha estimates from all form records were 0.775 (95% CI 0.520–0.863; *n* = 27), 0.827 (0.797–0.850; *n* = 235), and 0.835 (0.784–0.870; *n* = 175) for Exams 1–3. Corresponding case-subscale estimates were 0.613 (0.430–0.724), 0.807 (0.767–0.838), and 0.752 (0.694–0.798). The deduplicated full-form estimates were 0.775, 0.828, and 0.805, demonstrating that the analysis scope should accompany every reliability estimate. Item-level proportions correct and item-rest correlations are reported in Supplementary Table S7, and full-form/case-subscale estimates with denominators and uncertainty in Supplementary Table S8.

Only 21 of the 100 Exam 2 items met the minimum group-response requirements for the exploratory DIF models; 79 failed because at least one specialty had fewer than five correct or five incorrect responses. None of the 21 estimable items was flagged after the stated FDR correction (Supplementary Table S9). This finding applies only to the estimable subset and the specified matching models. It is not evidence that all items function equivalently across specialties.

### Exploratory score-gap matrix

Surgery had negative descriptive gaps for all four formats, including −8.2 percentage points for case analysis. Anesthesiology had a case-analysis gap of −7.4 points. Corrected case-analysis gaps were +6.4 for internal medicine, +7.1 for orthopedics, −1.9 for critical care medicine, and +6.0 for other clinical specialties (Table 3; Figure 2). These values locate observed differences relative to the same-form cohort mean; the display categories do not identify a clinical or educational deficit.

**Table 3:** Department mean minus the PGY-2 peer mean by question type (same-examination subgroup, *n* = 227; percentage points).

Department	A1	A2	A3/A4	Case analysis	Negative cells	Display category
Internal medicine	+2.6	+1.1	+1.6	+6.4	0	None
Surgery	−2.9	−2.5	−2.1	−8.2	4	Critical
Anesthesiology	+0.7	+0.9	−1.1	−7.4	2	High
Orthopedics	+2.5	+3.5	+2.3	+7.1	0	None
Critical care	+0.1	−4.6	−2.9	−1.9	3	Not assigned (*n* = 6)
Other clinical	+0.5	+1.3	+1.3	+6.0	0	None

PGY-2 overall means: A1, 76.6; A2, 86.3; A3/A4, 86.8; case analysis, 59.8 (all are percentages). Display labels use the retained heuristic cutoffs: critical (< −8), high (−8 to < −5), moderate (−5 to < −2), and low (−2 to < 0). These display categories do not prescribe an educational intervention and have not been validated. Every group contributes to the overall mean. Critical care *n* = 6; no display category is assigned because its estimates are particularly imprecise. Full specialty means, SDs, n, and 95% CIs are in Supplementary Table S5.

## Discussion

This single-center study found specialty-associated differences in ICU rotation exit examination scores among residents taking the same form at the same PGY. The primary ANOVA showed an association, and surgery had the lowest mean total score. These findings describe performance on the local examination at rotation exit. They do not establish inherent specialty differences, differences in clinical competence, or differential gains caused by the ICU rotation.

The complementary models showed that the magnitude and ranking of specialty associations depended on the score format, analytical approach, and classification. Within-form standardized scores describe relative position within each PGY/form and combine residents with different PGY compositions; they do not estimate the same comparison as raw scores restricted to PGY-2. The lowest standardized mean in anesthesiology therefore limits any claim that surgery consistently scored lowest. Merging orthopedics into Surgery also attenuated the association and changed the lowest-ranked group (Supplementary Table S10). The 21.3% total-score attenuation ratio describes the difference between two statistical quantities after conditioning on the combined PGY/form grouping. It does not isolate a pure PGY contribution. Similarly, the proximity of the crude and same-form *η*^2^ estimates cannot establish that form differences had little influence, because the analyses use different samples and do not test interchangeability of scores.

Case-analysis scores showed a larger between-specialty association than the other format scores in the same-form analysis. All-or-none multiple-response scoring may be sensitive to incomplete knowledge, but a larger group effect does not by itself demonstrate better item discrimination or a higher cognitive level (19). Learning-transfer distance was not measured, and these findings do not directly test the hypothesis (4, 5). Content-domain representation, item characteristics, and prior experience remain alternative explanations. The case-analysis regression pattern also illustrates suppression: conditioning on PGY/form increased the specialty contribution relative to the crude estimate.

Orthopedics residents scored above the same-form cohort mean across all four question types, whereas anesthesiology residents had a negative case-analysis gap despite the overlap between anesthesiology and ICU practice. These patterns do not support a simple ordering of written scores by presumed proximity to ICU work. Individual prior experience was not measured. Baseline knowledge or aptitude, supervision quality, case exposure, motivation, and differences between clinical competence and what the written examination measures are alternative explanations that this study cannot distinguish.

For program evaluation, comparisons should preferentially use a common form or a defensible score-linking procedure and should adjust for training stage before departments are ranked. Where multiple forms differ materially in difficulty or composition, within-form standardization alone does not provide formal equating (14). More broadly, contemporary critical care education increasingly favors multimodal, longitudinal assessment because no single written exit examination can capture the range of technical, reasoning, teamwork, and entrustment-related competencies expected in ICU practice (8, 10).

The gap matrix can inform hypotheses for curriculum review but should not be treated as proof of a training deficit. If prospective baseline-controlled work confirms that the surgery and anesthesiology case-analysis gaps emerge or widen during the ICU rotation, targeted case-based reasoning exercises followed by repeated assessment would be reasonable interventions to evaluate (20, 21). Until then, the matrix is best used to prioritize inquiry rather than to make high-stakes judgments about departments or trainees.

### Limitations

The single-center setting and locally developed examinations limit generalizability. No pre-rotation baseline was available, and previous ICU experience, supervision, case exposure, motivation, and other contextual factors were not measured (22). PGY and examination form were completely linked, so their independent effects cannot be estimated. No pilot testing, systematic blueprint development, or formal content-domain weight review was undertaken before administration. Cognitive-level mapping was unavailable, and evidence establishing cross-version score equating was not established. Differences in difficulty or content representation may therefore contribute to the observed associations; retrospective item analyses and reliability estimates do not resolve these validity limitations.

The exploratory DIF assessment was constrained by small specialty groups and sparse item-response cells: 79 of 100 Exam 2 items were not stably estimable under the stated rule. The absence of FDR flags among the other 21 items is insufficient to establish absence of DIF or measurement invariance (23, 24). Exam 1 estimates were particularly imprecise because *n* = 27. The PGY-2 critical care group had only six residents; anesthesiology and orthopedics had 20 and 21, respectively. The original Other Clinical coding combined heterogeneous categories, several with *n* = 1, and included one non-specialty status label. Source fields were incomplete for some records, including the retained record of one resident whose later records were coded as anesthesiology. Alternative classifications did not eliminate this historical uncertainty (Supplementary Tables S10, S11). The 437-record analysis includes repeated residents and is a descriptive reference, not an independent replication. The additional-record and first-versus-last-record comparisons cannot exclude selection bias. Finally, the gap matrix is exploratory. Written examination scores do not encompass clinical performance, teamwork, or entrustment.

## Conclusion

Specialty-associated differences in ICU exit examination performance were observed within this single-center educational setting and with the assessment instrument used here. Same-form analyses supported an association, but the results do not establish inherent differences between specialties or causal differences in rotation effectiveness. Prospective baseline-controlled studies using common-form or defensibly linked assessments and multimodal evaluation are needed before applying these patterns to specialty-targeted educational decisions (25, 26).

## Data Availability

The data analyzed in this study is subject to the following licenses/restrictions: The de-identified resident-level data and analysis code supporting the conclusions may be made available by the corresponding author upon reasonable request, subject to institutional and ethics approval. Examination items are not publicly available because disclosure could compromise assessment integrity. Requests to access these datasets should be directed to Lianmin Ye, ylmwork88@126.com.

## References

[1] National Health and Family Planning Commission of the People's Republic of China, and six other departments. Guiding Opinions on Establishing a Standardized Residency Training System. Beijing: National Health and Family Planning Commission (2014).

[2] Chinese Medical Doctor Association. Guideline for 360-Degree Evaluation in Standardized Residency Training. 2022 edn Beijing: Chinese Medical Doctor Association (2022).

[3] China Consortium of Elite Teaching Hospitals for Residency Education. Consensus on core competency framework for residency education among China Consortium of Elite Teaching Hospitals for Residency Education. Med J Peking Union Med Coll Hosp. (2022) 13:17–23. 10.12290/xhyxzz.2021-0755

[4] Mylopoulos M Brydges R Woods NN Manzone J Schwartz DL. Preparation for future learning: a missing competency in health professions education? Med Educ. (2016) 50:115–23. 10.1111/medu.12893 26695471

[5] Perkins DN Salomon G. Transfer of learning. In: Husén T Postlethwaite TN, editors. The International Encyclopedia of Education. Vol. 11. 2nd ed. Oxford: Pergamon (1994). p. 6452–57.

[6] Piquette D Goffi A Lee C Brydges R Walsh CM Mema B. Resident competencies before and after short intensive care unit rotations: a multicentre pilot observational study. Can J Anaesth. (2021) 68:235–44. 10.1007/s12630-020-01850-x 33174164

[7] Watari T Nishizaki Y Houchens N Kataoka K Sakaguchi K Shiraishi Y. Medical resident's pursuing specialty and differences in clinical proficiency among medical residents in Japan: a nationwide cross-sectional study. BMC Med Educ. (2023) 23:464. 10.1186/s12909-023-04429-4 37349724 PMC10286340

[8] Jaramillo-Villegas C Hernandez-Rincon EH Poveda-Henao CM Guzman-Sabogal YR. Assessment methods in graduate medical education in critical care: a scoping review. BMC Med Educ. (2026) 26:225. 10.1186/s12909-025-08451-6 41519723 PMC12888572

[9] Khan MF Aziz A Riaz Q Ghaffar WB Khan F Latif A. The impact of short intensive care rotation on residents’ experience in the COVID-19 pandemic: a mixed-methods study. J Med Educ Curric Dev. (2023) 10:23821205231222084. 10.1177/23821205231222084 38143724 PMC10748624

[10] Liu P Chen S-Y Chang Y-C Ng C-J Chaou C-H. Multimodal in-training examination in an emergency medicine residency training program: a longitudinal observational study. Front Med. (2022) 9:840721. 10.3389/fmed.2022.840721 PMC8959571 35355591

[11] Wu J Wang S Deng Y You N Zhang S Yu L. Course-specific performance and mentor characteristics in an undergraduate tutorial system in medical education. Front Med. (2026) 13:1862688. 10.3389/fmed.2026.1862688 PMC13429845 42548779

[12] Kinnear B Schumacher DJ Driessen EW Varpio L. How argumentation theory can inform assessment validity: a critical review. Med Educ. (2022) 56:1064–75. 10.1111/medu.14882 35851965 PMC9796688

[13] Görlich D Friederichs H. Using longitudinal progress test data to determine the effect size of learning in undergraduate medical education: a retrospective, single-center, mixed model analysis of progress testing results. Med Educ Online. (2021) 26:1972505. 10.1080/10872981.2021.1972505 PMC8409971 34459724

[14] Sun T Kim SY. Evaluating equating methods for varying levels of form difference. Educ Psychol Meas. (2024) 84:510–29. 10.1177/00131644231176989 38756465 PMC11095324

[15] von Elm E Altman DG Egger M Pocock SJ Gøtzsche PC Vandenbroucke JP. The Strengthening the Reporting of Observational Studies in Epidemiology (STROBE) statement: guidelines for reporting observational studies. Lancet. (2007) 370:1453–7. 10.1016/S0140-6736(07)61602-X 18064739

[16] Rezigalla AA. Item analysis: concept and application. In: Firstenberg MS Stawicki SP, editors. Medical Education for the 21st Century. London: IntechOpen (2022). p. 1–16. 10.5772/intechopen.100138

[17] Tavakol M Dennick R. Making sense of Cronbach's alpha. Int J Med Educ. (2011) 2:53–5. 10.5116/ijme.4dfb.8dfd 28029643 PMC4205511

[18] Cohen J. Statistical Power Analysis for the Behavioral Sciences. 2nd ed Hillsdale: Lawrence Erlbaum (1988).

[19] Miller GE. The assessment of clinical skills/competence/performance. Acad Med. (1990) 65:S63–7. 10.1097/00001888-199009000-00045 2400509

[20] Kern DE. Overview: a six-step approach to curriculum development. In: Thomas PA Kern DE Hughes MT Chen BY, editors. Curriculum Development for Medical Education: A Six-Step Approach. 3rd ed. Baltimore: Johns Hopkins University Press (2016). p. 5–10.

[21] Kirkpatrick DL Kirkpatrick JD. Evaluating Training Programs: The Four levels. 3rd ed San Francisco: Berrett-Koehler (2006).

[22] Conroy M McCallister J Gustin J. Entrustment decision making in the intensive care unit: it's about more than the learner. ATS Sch. (2024) 5:53–70. 10.34197/ats-scholar.2023-0060OC 38628300 PMC11019764

[23] Zwick R. A review of ETS differential item functioning assessment procedures: flagging rules, minimum sample size requirements, and criterion refinement. ETS Res Rep Ser. (2012) 2012:i–30. 10.1002/j.2333-8504.2012.tb02290.x

[24] Dale ED Abulela MAA Jia H Violato C. Are medical school preclinical tests biased for sex and race? A differential item functioning analysis. BMC Med Educ. (2025) 25:146. 10.1186/s12909-024-06540-6 39881271 PMC11780802

[25] Frank JR Snell LS Cate OT Holmboe ES Carraccio C Swing SR. Competency-based medical education: theory to practice. Med Teach. (2010) 32:638–45. 10.3109/0142159X.2010.501190 20662574

[26] Swanson DB Case SM. Trends in written assessment: a strangely biased perspective. In: Harden R Hart I Mulholland H, editors. Approaches to the Assessment of Clinical Competence. Part 1. Norwich: Page Brothers (1992). p. 38–53.

