# Supplementary material for: Specialty-associated differences in intensive care unit rotation exit examination performance: a single-center retrospective cross-sectional study
Source: Front Med (Lausanne). 2026 Oct 7;13:1960270. doi: 10.3389/fmed.2026.1960270 (PMC13683141; doi:10.3389/fmed.2026.1960270)
Supplement: Supplementary file 1 [file Supplementaryfile1.docx]

**Supplementary Material**

*Supplementary Tables S1–S11*

**Supplementary Table S1. STROBE checklist for cross-sectional studies**

Reporting items follow the STROBE statement for observational cross-sectional studies. Section names refer to the main manuscript.

| **Item** | **STROBE recommendation (abbreviated)** | **Addressed in** |
| --- | --- | --- |
| 1 | Title and abstract—informative title; balanced structured abstract | Title; Abstract |
| 2 | Introduction—background and rationale | Introduction (paragraphs 1–4) |
| 3 | Objectives and hypotheses | Introduction (final paragraph) |
| 4 | Study design | Materials and Methods—Study design and participants |
| 5 | Setting, locations, and dates | Materials and Methods—Study design and participants |
| 6 | Participants—eligibility and selection | Study design and participants; Department grouping |
| 7 | Variables—outcomes, predictors, and confounders | Examination instruments; Analytical strategy |
| 8 | Data sources and measurement | Examination instruments; Scored-item analyses and reliability; S6–S8 |
| 9 | Bias | Design limitation; Discussion—Limitations |
| 10 | Study size | Study design and participants (n = 412) |
| 11 | Quantitative variables and groupings | Department grouping; Analytical strategy |
| 12 | Statistical methods, confounding control, and sensitivity analyses | Analytical strategy; Gap matrix; Statistical software and use of generative AI |
| 13 | Participants—numbers at each stage | Results—Sample composition; Table 1 |
| 14 | Descriptive data | Results—Sample composition; Table 1; Supplementary Tables S2–S5 |
| 15 | Outcome data | Results; Tables 2–3 |
| 16 | Main results—estimates and confidence intervals | Results; Tables 1–2; S4–S5; Figures 1–3 |
| 17 | Other analyses—subgroups and sensitivity analyses | Results; Limitations; S3–S4; S9–S11 |
| 18 | Key results | Discussion (opening); Conclusion |
| 19 | Limitations | Design limitation; Discussion—Limitations |
| 20 | Interpretation | Discussion |
| 21 | Generalizability | Discussion—Limitations |
| 22 | Funding | Funding statement |

**Supplementary Table S2. Descriptive statistics for recorded categories within the original other clinical group (n = 78)**

| **Specialty** | **n** | **Mean** | **SD** | **95% CI** |
| --- | --- | --- | --- | --- |
| Neurology | 25 | 80.6 | 8.7 | 77.0–84.2 |
| Ultrasound medicine | 16 | 82.3 | 8.1 | 77.9–86.6 |
| Otorhinolaryngology | 12 | 82.6 | 8.3 | 77.3–87.9 |
| Psychiatry | 9 | 83.8 | 7.7 | 77.8–89.7 |
| Rehabilitation medicine | 7 | 85.1 | 5.6 | 80.0–90.3 |
| Radiation oncology | 3 | 77.0 | 5.3 | 63.9–90.1 |
| Radiotherapy | 2 | 93.5 | 4.9 | 49.0–138.0 |
| Infectious diseases | 1 | 80.0 | NE | NE |
| General practice | 1 | 87.0 | NE | NE |
| Plastic surgery | 1 | 84.0 | NE | NE |
| Other/unspecified | 1 | 82.0 | NE | NE |

SD, standard deviation; NE, not estimable. Total scores range from 0 to 100. The untruncated t interval for the n = 2 radiotherapy subgroup exceeds the score range, illustrating very low precision; it is not evidence of possible scores above 100. The exploratory ANOVA gave F(10,67) = 0.80, p = 0.625, η² = 0.107. Several subgroups are too small for reliable inference; this result does not establish homogeneity. The Other/unspecified record carried a status label rather than a specialty label; its available source department and specialty fields identify anesthesiology. The original coding is shown here for reference; a source-informed alternative is reported in S10.

**Supplementary Table S3. Same-form sensitivity analysis including all PGY-2 specialties with n ≥ 20 (n = 221)**

| **Specialty** | **n** | **Mean** | **SD** | **95% CI** |
| --- | --- | --- | --- | --- |
| Internal Medicine | 54 | 82.0 | 9.3 | 79.5–84.6 |
| Surgery | 77 | 75.9 | 9.1 | 73.9–78.0 |
| Anesthesiology | 20 | 78.1 | 7.6 | 74.5–81.7 |
| Orthopedics | 21 | 83.1 | 7.7 | 79.6–86.6 |
| Other Clinical | 49 | 81.5 | 8.4 | 79.1–83.9 |

ANOVA: F(4,216) = 6.03, η² = 0.100, p = 0.00013. Only critical care medicine (n = 6) was excluded. Orthopedics (n = 21) remained included under the original n ≥ 20 threshold.

**Supplementary Table S4. Specialty associations across complementary analytical descriptions**

| **Strategy** | **n** | **F** | **df** | **η²** | **p** | **Lowest mean** |
| --- | --- | --- | --- | --- | --- | --- |
| Crude total score | 412 | 8.65 | 5, 406 | 0.096 | <0.001 | Surgery |
| Same form / PGY-2 | 227 | 4.81 | 5, 221 | 0.098 | <0.001 | Surgery |
| Within-form standardized | 412 | 6.17 | 5, 406 | 0.071 | <0.001 | Anesthesiology |
| All-record reference (non-independent) | 437 | 7.70 | 5, 431 | 0.082 | <0.001 | Surgery |

The six Spearman correlations between the four specialty mean-ranking strategies were 0.771, 0.714, 1.000, 0.771, 0.771, and 0.714 (range 0.714–1.000). Considering only the three raw-total strategies gives a range of 0.771–1.000. Similar effect sizes across different cohorts or scales do not establish score interchangeability. Within-form z scores are not equated scores. The 437-record analysis includes repeated residents and is reported only for descriptive comparison.

*Supplementary Table S5. Group descriptives and primary contrasts*

Means, sample SDs and 95% t confidence intervals were calculated from unrounded data. Total scores use 0–100 points; A1/A2/A3–A4/case scores use percentages. Within-form standardized totals use z-score units. NE indicates an undefined estimate. The all-record group is a non-independent reference. The primary same-form cohort is PGY-2 n = 227; its ≥20-per-specialty subset is n = 221.

Exam 2 / PGY-2: six specialties (n = 227)

| **Specialty** | **Score** | **n** | **Mean** | **SD** | **95% CI** |
| --- | --- | --- | --- | --- | --- |
| Internal Medicine | total | 54 | 82.04 | 9.30 | 79.50–84.57 |
| Surgery | total | 77 | 75.92 | 9.06 | 73.87–77.98 |
| Anesthesiology | total | 20 | 78.10 | 7.62 | 74.53–81.67 |
| Orthopedics | total | 21 | 83.10 | 7.70 | 79.59–86.60 |
| Critical Care | total | 6 | 76.83 | 12.70 | 63.50–90.16 |
| Other Clinical | total | 49 | 81.51 | 8.40 | 79.10–83.92 |
| Internal Medicine | a1 | 54 | 79.17 | 10.58 | 76.28–82.06 |
| Surgery | a1 | 77 | 73.64 | 11.17 | 71.10–76.17 |
| Anesthesiology | a1 | 20 | 77.25 | 10.32 | 72.42–82.08 |
| Orthopedics | a1 | 21 | 79.05 | 10.08 | 74.46–83.63 |
| Critical Care | a1 | 6 | 76.67 | 6.06 | 70.31–83.02 |
| Other Clinical | a1 | 49 | 77.04 | 11.45 | 73.75–80.33 |
| Internal Medicine | a2 | 54 | 87.41 | 11.14 | 84.37–90.45 |
| Surgery | a2 | 77 | 83.81 | 9.05 | 81.76–85.86 |
| Anesthesiology | a2 | 20 | 87.17 | 9.06 | 82.93–91.41 |
| Orthopedics | a2 | 21 | 89.83 | 8.67 | 85.88–93.77 |
| Critical Care | a2 | 6 | 81.67 | 11.50 | 69.60–93.73 |
| Other Clinical | a2 | 49 | 87.62 | 8.98 | 85.04–90.20 |
| Internal Medicine | a34 | 54 | 88.41 | 7.64 | 86.32–90.49 |
| Surgery | a34 | 77 | 84.66 | 8.57 | 82.71–86.60 |
| Anesthesiology | a34 | 20 | 85.64 | 9.57 | 81.16–90.12 |
| Orthopedics | a34 | 21 | 89.09 | 7.53 | 85.66–92.51 |
| Critical Care | a34 | 6 | 83.85 | 12.92 | 70.29–97.41 |
| Other Clinical | a34 | 49 | 88.08 | 7.71 | 85.86–90.29 |
| Internal Medicine | case | 54 | 66.18 | 20.26 | 60.65–71.71 |
| Surgery | case | 77 | 51.61 | 23.38 | 46.30–56.91 |
| Anesthesiology | case | 20 | 52.37 | 16.34 | 44.72–60.01 |
| Orthopedics | case | 21 | 66.91 | 29.35 | 53.55–80.27 |
| Critical Care | case | 6 | 57.90 | 24.91 | 31.76–84.04 |
| Other Clinical | case | 49 | 65.84 | 21.27 | 59.73–71.95 |

*All unique residents (n = 412)*

| **Specialty** | **Score** | **n** | **Mean** | **SD** | **95% CI** |
| --- | --- | --- | --- | --- | --- |
| Internal Medicine | total | 156 | 82.98 | 7.99 | 81.72–84.24 |
| Surgery | total | 98 | 76.86 | 8.76 | 75.10–78.61 |
| Anesthesiology | total | 31 | 77.23 | 8.02 | 74.28–80.17 |
| Orthopedics | total | 29 | 82.31 | 8.24 | 79.18–85.45 |
| Critical Care | total | 20 | 80.55 | 8.29 | 76.67–84.43 |
| Other Clinical | total | 78 | 82.33 | 7.93 | 80.55–84.12 |
| Internal Medicine | a1 | 156 | 86.41 | 11.05 | 84.66–88.16 |
| Surgery | a1 | 98 | 77.11 | 12.81 | 74.54–79.68 |
| Anesthesiology | a1 | 31 | 78.65 | 13.01 | 73.88–83.43 |
| Orthopedics | a1 | 29 | 82.12 | 10.72 | 78.05–86.20 |
| Critical Care | a1 | 20 | 85.51 | 8.00 | 81.76–89.25 |
| Other Clinical | a1 | 78 | 81.84 | 11.84 | 79.17–84.51 |
| Internal Medicine | a2 | 156 | 88.98 | 8.51 | 87.63–90.32 |
| Surgery | a2 | 98 | 84.61 | 8.93 | 82.82–86.40 |
| Anesthesiology | a2 | 31 | 87.21 | 8.32 | 84.15–90.26 |
| Orthopedics | a2 | 29 | 88.44 | 9.67 | 84.76–92.12 |
| Critical Care | a2 | 20 | 83.41 | 9.56 | 78.94–87.88 |
| Other Clinical | a2 | 78 | 87.99 | 8.81 | 86.01–89.98 |
| Internal Medicine | a34 | 156 | 88.46 | 7.65 | 87.25–89.67 |
| Surgery | a34 | 98 | 84.82 | 8.29 | 83.16–86.48 |
| Anesthesiology | a34 | 31 | 84.64 | 9.12 | 81.29–87.98 |
| Orthopedics | a34 | 29 | 87.67 | 9.71 | 83.98–91.37 |
| Critical Care | a34 | 20 | 86.37 | 7.88 | 82.68–90.05 |
| Other Clinical | a34 | 78 | 88.41 | 7.20 | 86.79–90.04 |
| Internal Medicine | case | 156 | 61.39 | 20.51 | 58.14–64.63 |
| Surgery | case | 98 | 51.25 | 22.22 | 46.79–55.70 |
| Anesthesiology | case | 31 | 47.98 | 16.53 | 41.92–54.04 |
| Orthopedics | case | 29 | 62.66 | 28.23 | 51.92–73.40 |
| Critical Care | case | 20 | 53.04 | 21.88 | 42.80–63.28 |
| Other Clinical | case | 78 | 63.06 | 22.11 | 58.08–68.05 |

Exam 2 / PGY-2: five specialties with n ≥ 20 (n = 221)

| **Specialty** | **Score** | **n** | **Mean** | **SD** | **95% CI** |
| --- | --- | --- | --- | --- | --- |
| Internal Medicine | total | 54 | 82.04 | 9.30 | 79.50–84.57 |
| Surgery | total | 77 | 75.92 | 9.06 | 73.87–77.98 |
| Anesthesiology | total | 20 | 78.10 | 7.62 | 74.53–81.67 |
| Orthopedics | total | 21 | 83.10 | 7.70 | 79.59–86.60 |
| Other Clinical | total | 49 | 81.51 | 8.40 | 79.10–83.92 |

All examination records: descriptive reference (n = 437)

| **Specialty** | **Score** | **n** | **Mean** | **SD** | **95% CI** |
| --- | --- | --- | --- | --- | --- |
| Internal Medicine | total | 159 | 82.63 | 8.56 | 81.29–83.97 |
| Surgery | total | 98 | 76.86 | 8.76 | 75.10–78.61 |
| Anesthesiology | total | 46 | 77.74 | 8.34 | 75.26–80.22 |
| Orthopedics | total | 29 | 82.31 | 8.24 | 79.18–85.45 |
| Critical Care | total | 27 | 81.33 | 8.22 | 78.08–84.58 |
| Other Clinical | total | 78 | 82.33 | 7.93 | 80.55–84.12 |
| Internal Medicine | a1 | 159 | 86.35 | 10.99 | 84.63–88.07 |
| Surgery | a1 | 98 | 77.11 | 12.81 | 74.54–79.68 |
| Anesthesiology | a1 | 46 | 80.62 | 12.08 | 77.03–84.20 |
| Orthopedics | a1 | 29 | 82.12 | 10.72 | 78.05–86.20 |
| Critical Care | a1 | 27 | 84.45 | 9.48 | 80.70–88.20 |
| Other Clinical | a1 | 78 | 81.84 | 11.84 | 79.17–84.51 |
| Internal Medicine | a2 | 159 | 88.74 | 8.98 | 87.34–90.15 |
| Surgery | a2 | 98 | 84.61 | 8.93 | 82.82–86.40 |
| Anesthesiology | a2 | 46 | 86.67 | 8.70 | 84.08–89.25 |
| Orthopedics | a2 | 29 | 88.44 | 9.67 | 84.76–92.12 |
| Critical Care | a2 | 27 | 84.62 | 9.06 | 81.04–88.21 |
| Other Clinical | a2 | 78 | 87.99 | 8.81 | 86.01–89.98 |
| Internal Medicine | a34 | 159 | 87.97 | 8.80 | 86.60–89.35 |
| Surgery | a34 | 98 | 84.82 | 8.29 | 83.16–86.48 |
| Anesthesiology | a34 | 46 | 85.05 | 9.10 | 82.35–87.76 |
| Orthopedics | a34 | 29 | 87.67 | 9.71 | 83.98–91.37 |
| Critical Care | a34 | 27 | 87.02 | 7.44 | 84.08–89.97 |
| Other Clinical | a34 | 78 | 88.41 | 7.20 | 86.79–90.04 |
| Internal Medicine | case | 159 | 60.89 | 20.84 | 57.63–64.15 |
| Surgery | case | 98 | 51.25 | 22.22 | 46.79–55.70 |
| Anesthesiology | case | 46 | 48.81 | 20.84 | 42.62–55.00 |
| Orthopedics | case | 29 | 62.66 | 28.23 | 51.92–73.40 |
| Critical Care | case | 27 | 57.23 | 22.62 | 48.28–66.18 |
| Other Clinical | case | 78 | 63.06 | 22.11 | 58.08–68.05 |

*Within-form standardized total scores (n = 412)*

| **Specialty** | **Score** | **n** | **Mean** | **SD** | **95% CI** |
| --- | --- | --- | --- | --- | --- |
| Internal Medicine | z_total | 156 | 0.19 | 0.99 | 0.04–0.35 |
| Surgery | z_total | 98 | -0.36 | 0.96 | -0.56–-0.17 |
| Anesthesiology | z_total | 31 | -0.42 | 1.02 | -0.80–-0.04 |
| Orthopedics | z_total | 29 | 0.21 | 1.01 | -0.18–0.59 |
| Critical Care | z_total | 20 | -0.13 | 0.92 | -0.56–0.30 |
| Other Clinical | z_total | 78 | 0.20 | 0.91 | -0.01–0.41 |

*PGY-1*

| **Specialty** | **Score** | **n** | **Mean** | **SD** | **95% CI** |
| --- | --- | --- | --- | --- | --- |
| Internal Medicine | total | 1 | 85.00 | NE | NE |
| Surgery | total | 2 | 83.50 | 13.44 | -37.21–204.21 |
| Anesthesiology | total | 1 | 80.00 | NE | NE |
| Orthopedics | total | 2 | 75.50 | 20.51 | -108.74–259.74 |
| Critical Care | total | 11 | 82.91 | 5.80 | 79.01–86.81 |
| Other Clinical | total | 10 | 83.80 | 7.28 | 78.59–89.01 |
| Internal Medicine | a1 | 1 | 90.00 | NE | NE |
| Surgery | a1 | 2 | 88.35 | 11.81 | -17.75–194.45 |
| Anesthesiology | a1 | 1 | 83.30 | NE | NE |
| Orthopedics | a1 | 2 | 83.30 | 14.14 | -43.76–210.36 |
| Critical Care | a1 | 11 | 88.19 | 4.29 | 85.31–91.08 |
| Other Clinical | a1 | 10 | 87.35 | 4.92 | 83.83–90.87 |
| Internal Medicine | a2 | 1 | 80.00 | NE | NE |
| Surgery | a2 | 2 | 86.65 | 18.88 | -82.98–256.28 |
| Anesthesiology | a2 | 1 | 90.00 | NE | NE |
| Orthopedics | a2 | 2 | 81.65 | 16.48 | -66.38–229.68 |
| Critical Care | a2 | 11 | 83.93 | 9.98 | 77.22–90.63 |
| Other Clinical | a2 | 10 | 83.01 | 10.82 | 75.27–90.75 |
| Internal Medicine | a34 | 1 | 96.60 | NE | NE |
| Surgery | a34 | 2 | 89.65 | 4.88 | 45.81–133.49 |
| Anesthesiology | a34 | 1 | 79.30 | NE | NE |
| Orthopedics | a34 | 2 | 72.40 | 29.27 | -190.62–335.42 |
| Critical Care | a34 | 11 | 87.79 | 5.19 | 84.30–91.28 |
| Other Clinical | a34 | 10 | 88.30 | 6.75 | 83.47–93.13 |
| Internal Medicine | case | 1 | 54.50 | NE | NE |
| Surgery | case | 2 | 45.45 | 25.67 | -185.17–276.07 |
| Anesthesiology | case | 1 | 45.50 | NE | NE |
| Orthopedics | case | 2 | 45.45 | 25.67 | -185.17–276.07 |
| Critical Care | case | 11 | 52.89 | 22.91 | 37.50–68.28 |
| Other Clinical | case | 10 | 64.54 | 25.13 | 46.56–82.52 |

PGY-3

| **Specialty** | **Score** | **n** | **Mean** | **SD** | **95% CI** |
| --- | --- | --- | --- | --- | --- |
| Internal Medicine | total | 101 | 83.47 | 7.24 | 82.04–84.90 |
| Surgery | total | 19 | 79.95 | 6.20 | 76.96–82.93 |
| Anesthesiology | total | 10 | 75.20 | 9.21 | 68.61–81.79 |
| Orthopedics | total | 6 | 81.83 | 6.15 | 75.38–88.28 |
| Critical Care | total | 3 | 79.33 | 2.52 | 73.08–85.58 |
| Other Clinical | total | 19 | 83.68 | 7.01 | 80.31–87.06 |
| Internal Medicine | a1 | 101 | 90.25 | 9.31 | 88.41–92.09 |
| Surgery | a1 | 19 | 90.00 | 10.41 | 84.98–95.02 |
| Anesthesiology | a1 | 10 | 81.00 | 18.07 | 68.07–93.93 |
| Orthopedics | a1 | 6 | 92.50 | 5.24 | 87.00–98.00 |
| Critical Care | a1 | 3 | 93.33 | 7.64 | 74.36–112.31 |
| Other Clinical | a1 | 19 | 91.32 | 7.97 | 87.48–95.16 |
| Internal Medicine | a2 | 101 | 89.90 | 6.59 | 88.60–91.20 |
| Surgery | a2 | 19 | 87.63 | 7.14 | 84.19–91.07 |
| Anesthesiology | a2 | 10 | 87.00 | 7.53 | 81.61–92.39 |
| Orthopedics | a2 | 6 | 85.83 | 11.58 | 73.68–97.99 |
| Critical Care | a2 | 3 | 85.00 | 5.00 | 72.58–97.42 |
| Other Clinical | a2 | 19 | 91.58 | 5.54 | 88.91–94.25 |
| Internal Medicine | a34 | 101 | 88.41 | 7.69 | 86.89–89.93 |
| Surgery | a34 | 19 | 84.97 | 7.50 | 81.36–88.58 |
| Anesthesiology | a34 | 10 | 83.16 | 8.70 | 76.93–89.39 |
| Orthopedics | a34 | 6 | 87.82 | 5.33 | 82.22–93.41 |
| Critical Care | a34 | 3 | 86.17 | 2.83 | 79.14–93.19 |
| Other Clinical | a34 | 19 | 89.34 | 6.26 | 86.33–92.36 |
| Internal Medicine | case | 101 | 58.89 | 20.38 | 54.87–62.91 |
| Surgery | case | 19 | 50.42 | 17.65 | 41.91–58.92 |
| Anesthesiology | case | 10 | 39.46 | 14.93 | 28.78–50.14 |
| Orthopedics | case | 6 | 53.50 | 24.32 | 27.98–79.02 |
| Critical Care | case | 3 | 43.87 | 13.24 | 10.98–76.75 |
| Other Clinical | case | 19 | 55.13 | 21.93 | 44.55–65.70 |

*Primary Tukey HSD contrasts*

| **First minus second specialty** | **Difference** | **Simultaneous 95% CI** | **Adjusted p** |
| --- | --- | --- | --- |
| Surgery-Internal Medicine | -6.11 | -10.63–-1.60 | 0.002 |
| Anesthesiology-Internal Medicine | -3.94 | -10.60–2.72 | 0.533 |
| Orthopedics-Internal Medicine | 1.06 | -5.48–7.60 | 0.997 |
| Critical Care-Internal Medicine | -5.20 | -16.15–5.74 | 0.747 |
| Other Clinical-Internal Medicine | -0.53 | -5.55–4.49 | 1.000 |
| Anesthesiology-Surgery | 2.18 | -4.21–8.56 | 0.924 |
| Orthopedics-Surgery | 7.17 | 0.91–13.44 | 0.014 |
| Critical Care-Surgery | 0.91 | -9.87–11.69 | 1.000 |
| Other Clinical-Surgery | 5.59 | 0.94–10.24 | 0.009 |
| Orthopedics-Anesthesiology | 5.00 | -2.95–12.94 | 0.463 |
| Critical Care-Anesthesiology | -1.27 | -13.11–10.57 | 1.000 |
| Other Clinical-Anesthesiology | 3.41 | -3.34–10.16 | 0.695 |
| Critical Care-Orthopedics | -6.26 | -18.04–5.51 | 0.646 |
| Other Clinical-Orthopedics | -1.59 | -8.22–5.05 | 0.983 |
| Other Clinical-Critical Care | 4.68 | -6.32–15.68 | 0.826 |

The primary PGY-2 ANOVA gave F(5,221) = 4.81, p = 0.00034, η² = 0.098. The median-centered Levene/Brown–Forsythe diagnostic gave F(5,221) = 0.94, p = 0.455. Shapiro–Wilk statistics are diagnostic rather than proof of normality.

| **Specialty** | **n** | **Shapiro W** | **p** |
| --- | --- | --- | --- |
| Internal Medicine | 54 | 0.967 | 0.138 |
| Surgery | 77 | 0.936 | <0.001 |
| Anesthesiology | 20 | 0.976 | 0.873 |
| Orthopedics | 21 | 0.949 | 0.325 |
| Critical Care | 6 | 0.929 | 0.571 |
| Other Clinical | 49 | 0.960 | 0.095 |

*Overall cohort and examination-form descriptives*

These rows supplement the specialty-level descriptions above. Within-form z-score totals are in standardized units; raw totals are in points and format scores in percentages. The 437-record and all-form-record rows include repeated residents; their ordinary t intervals are descriptive reference intervals that do not account for within-resident dependence. They are not used for independent inferential confirmation.

| **Cohort / form** | **Score** | **n** | **Mean** | **SD** | **95% CI** |
| --- | --- | --- | --- | --- | --- |
| All 412 residents | total | 412 | 80.80 | 8.58 | 79.97–81.63 |
| All 412 residents | a1 | 412 | 82.40 | 12.19 | 81.22–83.58 |
| All 412 residents | a2 | 412 | 87.31 | 8.95 | 86.44–88.18 |
| All 412 residents | a34 | 412 | 87.14 | 8.13 | 86.35–87.93 |
| All 412 residents | case | 412 | 57.97 | 22.22 | 55.82–60.12 |
| Exam 2 / PGY-2 (227) | total | 227 | 79.46 | 9.21 | 78.26–80.67 |
| Exam 2 / PGY-2 (227) | a1 | 227 | 76.59 | 10.96 | 75.15–78.02 |
| Exam 2 / PGY-2 (227) | a2 | 227 | 86.29 | 9.74 | 85.01–87.56 |
| Exam 2 / PGY-2 (227) | a34 | 227 | 86.76 | 8.42 | 85.66–87.86 |
| Exam 2 / PGY-2 (227) | case | 227 | 59.80 | 23.21 | 56.76–62.83 |
| Exam 2 five groups (221) | total | 221 | 79.53 | 9.13 | 78.32–80.74 |
| Exam 2 five groups (221) | a1 | 221 | 76.58 | 11.07 | 75.12–78.05 |
| Exam 2 five groups (221) | a2 | 221 | 86.41 | 9.69 | 85.13–87.69 |
| Exam 2 five groups (221) | a34 | 221 | 86.84 | 8.29 | 85.74–87.94 |
| Exam 2 five groups (221) | case | 221 | 59.85 | 23.22 | 56.77–62.93 |
| All 437 records | total | 437 | 80.67 | 8.75 | 79.84–81.49 |
| All 437 records | a1 | 437 | 82.47 | 12.06 | 81.34–83.61 |
| All 437 records | a2 | 437 | 87.19 | 9.08 | 86.34–88.04 |
| All 437 records | a34 | 437 | 86.96 | 8.52 | 86.16–87.76 |
| All 437 records | case | 437 | 57.74 | 22.55 | 55.62–59.86 |
| Within-form z (412) | z_total | 412 | 0.00 | 1.00 | -0.10–0.10 |
| Exam 1 / 27 residents | total | 27 | 82.70 | 7.71 | 79.65–85.75 |
| Exam 1 / 27 residents | a1 | 27 | 87.41 | 5.57 | 85.21–89.62 |
| Exam 1 / 27 residents | a2 | 27 | 83.70 | 10.31 | 79.62–87.78 |
| Exam 1 / 27 residents | a34 | 27 | 86.99 | 9.15 | 83.37–90.61 |
| Exam 1 / 27 residents | case | 27 | 55.89 | 22.91 | 46.83–64.95 |
| Exam 2 / 227 residents | total | 227 | 79.46 | 9.21 | 78.26–80.67 |
| Exam 2 / 227 residents | a1 | 227 | 76.59 | 10.96 | 75.15–78.02 |
| Exam 2 / 227 residents | a2 | 227 | 86.29 | 9.74 | 85.01–87.56 |
| Exam 2 / 227 residents | a34 | 227 | 86.76 | 8.42 | 85.66–87.86 |
| Exam 2 / 227 residents | case | 227 | 59.80 | 23.21 | 56.76–62.83 |
| Exam 3 / 158 residents | total | 158 | 82.41 | 7.40 | 81.24–83.57 |
| Exam 3 / 158 residents | a1 | 158 | 89.91 | 10.06 | 88.33–91.49 |
| Exam 3 / 158 residents | a2 | 158 | 89.40 | 6.87 | 88.32–90.48 |
| Exam 3 / 158 residents | a34 | 158 | 87.71 | 7.54 | 86.53–88.90 |
| Exam 3 / 158 residents | case | 158 | 55.70 | 20.46 | 52.48–58.91 |
| Exam 1 / 27 records | total | 27 | 82.70 | 7.71 | 79.65–85.75 |
| Exam 1 / 27 records | a1 | 27 | 87.41 | 5.57 | 85.21–89.62 |
| Exam 1 / 27 records | a2 | 27 | 83.70 | 10.31 | 79.62–87.78 |
| Exam 1 / 27 records | a34 | 27 | 86.99 | 9.15 | 83.37–90.61 |
| Exam 1 / 27 records | case | 27 | 55.89 | 22.91 | 46.83–64.95 |
| Exam 2 / 235 records | total | 235 | 79.53 | 9.18 | 78.35–80.71 |
| Exam 2 / 235 records | a1 | 235 | 76.66 | 10.96 | 75.25–78.07 |
| Exam 2 / 235 records | a2 | 235 | 86.28 | 9.69 | 85.04–87.53 |
| Exam 2 / 235 records | a34 | 235 | 86.77 | 8.35 | 85.70–87.85 |
| Exam 2 / 235 records | case | 235 | 60.07 | 23.19 | 57.09–63.05 |
| Exam 3 / 175 records | total | 175 | 81.87 | 8.11 | 80.66–83.08 |
| Exam 3 / 175 records | a1 | 175 | 89.51 | 9.95 | 88.03–91.00 |
| Exam 3 / 175 records | a2 | 175 | 88.94 | 7.63 | 87.80–90.08 |
| Exam 3 / 175 records | a34 | 175 | 87.20 | 8.70 | 85.91–88.50 |
| Exam 3 / 175 records | case | 175 | 54.89 | 21.35 | 51.71–58.08 |

Adjusted specialty means from the existing additive models

For each of the five outcomes, the additive model includes specialty and categorical PGY. Adjusted means average model predictions across PGY with weights 27/412, 227/412, and 158/412. They therefore refer to the full cohort training-stage/form distribution, which differs from each specialty’s observed distribution. The model n is 412; observed specialty n is shown for context. SE and 95% t CI quantify model uncertainty. Raw SDs are reported in the preceding descriptive tables. PGY and form are inseparable, so adjustment does not isolate their independent effects or equate scores.

| **Specialty** | **Score** | **Observed n** | **Adjusted mean** | **SE** | **95% CI** |
| --- | --- | --- | --- | --- | --- |
| Internal Medicine | total | 156 | 82.74 | 0.70 | 81.36–84.12 |
| Surgery | total | 98 | 77.28 | 0.85 | 75.60–78.95 |
| Anesthesiology | total | 31 | 77.41 | 1.47 | 74.52–80.31 |
| Orthopedics | total | 29 | 82.57 | 1.53 | 79.57–85.58 |
| Critical Care | total | 20 | 79.56 | 2.04 | 75.55–83.57 |
| Other Clinical | total | 78 | 82.38 | 0.94 | 80.53–84.22 |
| Internal Medicine | a1 | 156 | 83.73 | 0.88 | 82.00–85.47 |
| Surgery | a1 | 98 | 79.89 | 1.07 | 77.79–81.99 |
| Anesthesiology | a1 | 31 | 79.73 | 1.85 | 76.10–83.37 |
| Orthopedics | a1 | 29 | 84.27 | 1.92 | 80.50–88.04 |
| Critical Care | a1 | 20 | 83.65 | 2.56 | 78.62–88.69 |
| Other Clinical | a1 | 78 | 82.95 | 1.18 | 80.63–85.27 |
| Internal Medicine | a2 | 156 | 88.20 | 0.75 | 86.72–89.67 |
| Surgery | a2 | 98 | 84.96 | 0.91 | 83.18–86.75 |
| Anesthesiology | a2 | 31 | 87.28 | 1.57 | 84.19–90.36 |
| Orthopedics | a2 | 29 | 88.87 | 1.63 | 85.67–92.08 |
| Critical Care | a2 | 20 | 85.11 | 2.17 | 80.84–89.39 |
| Other Clinical | a2 | 78 | 88.48 | 1.00 | 86.51–90.45 |
| Internal Medicine | a34 | 156 | 88.45 | 0.69 | 87.10–89.81 |
| Surgery | a34 | 98 | 84.81 | 0.84 | 83.17–86.46 |
| Anesthesiology | a34 | 31 | 84.63 | 1.45 | 81.79–87.47 |
| Orthopedics | a34 | 29 | 87.67 | 1.50 | 84.72–90.62 |
| Critical Care | a34 | 20 | 86.45 | 2.00 | 82.52–90.39 |
| Other Clinical | a34 | 78 | 88.43 | 0.92 | 86.61–90.24 |
| Internal Medicine | case | 156 | 63.16 | 1.84 | 59.54–66.78 |
| Surgery | case | 98 | 49.58 | 2.23 | 45.19–53.97 |
| Anesthesiology | case | 31 | 47.36 | 3.86 | 39.78–54.94 |
| Orthopedics | case | 29 | 61.31 | 4.00 | 53.44–69.18 |
| Critical Care | case | 20 | 53.42 | 5.34 | 42.91–63.92 |
| Other Clinical | case | 78 | 62.27 | 2.46 | 57.42–67.11 |

Regression coefficients for the existing additive models

All models use 412 residents. Reference categories are internal medicine and PGY-1 / Exam 1. Specialty coefficients compare scores with internal medicine conditional on PGY/form; PGY coefficients compare the combined PGY/form groups. The intercept is the predicted internal-medicine PGY-1 score. Intervals are model-based 95% t CIs with 404 residual degrees of freedom; they are not simultaneous multiple-comparison intervals.

| **Score** | **Term** | **Coefficient** | **SE** | **95% CI** |
| --- | --- | --- | --- | --- |
| total | Intercept | 84.74 | 1.98 | 80.85–88.64 |
| total | PGY-2 / Exam 2 | -2.78 | 1.88 | -6.47–0.90 |
| total | PGY-3 / Exam 3 | -1.24 | 1.96 | -5.10–2.63 |
| total | Surgery | -5.46 | 1.13 | -7.69–-3.23 |
| total | Anesthesiology | -5.32 | 1.64 | -8.54–-2.11 |
| total | Orthopedics | -0.16 | 1.70 | -3.51–3.19 |
| total | Critical care | -3.17 | 2.21 | -7.52–1.17 |
| total | Other clinical | -0.36 | 1.21 | -2.73–2.01 |
| a1 | Intercept | 88.13 | 2.49 | 83.23–93.02 |
| a1 | PGY-2 / Exam 2 | -9.77 | 2.36 | -14.40–-5.13 |
| a1 | PGY-3 / Exam 3 | 2.57 | 2.47 | -2.29–7.42 |
| a1 | Surgery | -3.84 | 1.42 | -6.64–-1.04 |
| a1 | Anesthesiology | -4.00 | 2.05 | -8.04–0.04 |
| a1 | Orthopedics | 0.54 | 2.14 | -3.67–4.74 |
| a1 | Critical care | -0.08 | 2.78 | -5.54–5.38 |
| a1 | Other clinical | -0.78 | 1.51 | -3.76–2.20 |
| a2 | Intercept | 85.08 | 2.11 | 80.92–89.23 |
| a2 | PGY-2 / Exam 2 | 2.35 | 2.00 | -1.59–6.28 |
| a2 | PGY-3 / Exam 3 | 4.77 | 2.09 | 0.65–8.89 |
| a2 | Surgery | -3.23 | 1.21 | -5.61–-0.86 |
| a2 | Anesthesiology | -0.92 | 1.74 | -4.35–2.51 |
| a2 | Orthopedics | 0.68 | 1.82 | -2.89–4.25 |
| a2 | Critical care | -3.08 | 2.36 | -7.72–1.55 |
| a2 | Other clinical | 0.28 | 1.29 | -2.24–2.81 |
| a34 | Intercept | 88.28 | 1.95 | 84.46–92.11 |
| a34 | PGY-2 / Exam 2 | 0.18 | 1.84 | -3.44–3.80 |
| a34 | PGY-3 / Exam 3 | 0.18 | 1.93 | -3.61–3.97 |
| a34 | Surgery | -3.64 | 1.11 | -5.83–-1.45 |
| a34 | Anesthesiology | -3.82 | 1.61 | -6.98–-0.67 |
| a34 | Orthopedics | -0.78 | 1.67 | -4.07–2.51 |
| a34 | Critical care | -2.00 | 2.17 | -6.27–2.27 |
| a34 | Other clinical | -0.03 | 1.18 | -2.35–2.30 |
| case | Intercept | 61.91 | 5.19 | 51.70–72.13 |
| case | PGY-2 / Exam 2 | 4.50 | 4.92 | -5.17–14.16 |
| case | PGY-3 / Exam 3 | -3.22 | 5.15 | -13.34–6.90 |
| case | Surgery | -13.58 | 2.97 | -19.42–-7.73 |
| case | Anesthesiology | -15.80 | 4.29 | -24.22–-7.37 |
| case | Orthopedics | -1.85 | 4.46 | -10.62–6.93 |
| case | Critical care | -9.74 | 5.79 | -21.13–1.65 |
| case | Other clinical | -0.89 | 3.16 | -7.10–5.32 |

*Supplementary Table S6. Examination structure and case-item options*

| **Form / PGY** | **Total items** | **Minutes** | **A1** | **A2** | **A3/A4** | **Case** |
| --- | --- | --- | --- | --- | --- | --- |
| Exam 1 / PGY-1 | 100 | 60 | 30 | 30 | 29 | 11 |
| Exam 2 / PGY-2 | 100 | 60 | 20 | 30 | 31 | 19 |
| Exam 3 / PGY-3 | 100 | 60 | 20 | 20 | 41 | 19 |

All items are worth 1 point. Case-analysis items use all-or-none scoring; there is no partial credit. Exams 1–3 correspond to ‘ICU Rotation Exit Examination 1,’ ‘ICU Rotation Exit Examination 2,’ and ‘ICU Rotation Exit Examination 3’ and were administered to residents completing their ICU rotation. Six consecutive monthly batches ran from November 29, 2025, to April 29, 2026. The six regular examination dates were November 29 and December 27, 2025, and January 29, February 27, March 27, and April 29, 2026. Individual resits were arranged separately. Every examination had 100 items and a 60-minute time limit. No pilot testing, systematic blueprint development, or formal content-domain weight review was undertaken. Cognitive-level mapping was unavailable. Item-format proportions are not content-domain weights. Formal score-equating evidence was not established; equal length and time limits do not establish psychometric equivalence. S7–S9 report retrospective revision analyses, not pre-administration validation.

Version-specific administration coverage in the available register is reported below. All three versions have dated entries from November 29, 2025, through April 29, 2026. Counts describe the administrative register only, which contains 381 scored entries (26, 201, and 154 for Exams 1–3); it is not the complete 437-record analysis file. A zero means no entry in this register, not confirmed non-administration. Two scored entries lack start dates. The analysis denominators remain 412 unique residents and 437 records.

| **Date** | **Exam 1 / PGY-1** | **Exam 2 / PGY-2** | **Exam 3 / PGY-3** |
| --- | --- | --- | --- |
| 2025-11-29 | 4 | 32 | 27 |
| 2025-12-27 | 12 | 27 | 22 |
| 2026-01-29 | 5 | 50 | 17 |
| 2026-02-27 | 1 | 26 | 42 |
| 2026-03-27 | 0 | 35 | 24 |
| 2026-04-29 | 4 | 30 | 21 |
| Start date missing | 0 | 1 | 1 |
| Total scored register entries | 26 | 201 | 154 |

| **Form** | **Item** | **Number of response options** |
| --- | --- | --- |
| Exam 1 | Q90 | 7 |
| Exam 1 | Q91 | 6 |
| Exam 1 | Q92 | 9 |
| Exam 1 | Q93 | 6 |
| Exam 1 | Q94 | 6 |
| Exam 1 | Q95 | 7 |
| Exam 1 | Q96 | 7 |
| Exam 1 | Q97 | 8 |
| Exam 1 | Q98 | 9 |
| Exam 1 | Q99 | 7 |
| Exam 1 | Q100 | 9 |
| Exam 2 | Q82 | 7 |
| Exam 2 | Q83 | 8 |
| Exam 2 | Q84 | 7 |
| Exam 2 | Q85 | 6 |
| Exam 2 | Q86 | 6 |
| Exam 2 | Q87 | 6 |
| Exam 2 | Q88 | 6 |
| Exam 2 | Q89 | 5 |
| Exam 2 | Q90 | 6 |
| Exam 2 | Q91 | 7 |
| Exam 2 | Q92 | 6 |
| Exam 2 | Q93 | 6 |
| Exam 2 | Q94 | 6 |
| Exam 2 | Q95 | 7 |
| Exam 2 | Q96 | 7 |
| Exam 2 | Q97 | 8 |
| Exam 2 | Q98 | 9 |
| Exam 2 | Q99 | 6 |
| Exam 2 | Q100 | 6 |
| Exam 3 | Q82 | 6 |
| Exam 3 | Q83 | 6 |
| Exam 3 | Q84 | 6 |
| Exam 3 | Q85 | 7 |
| Exam 3 | Q86 | 7 |
| Exam 3 | Q87 | 8 |
| Exam 3 | Q88 | 7 |
| Exam 3 | Q89 | 9 |
| Exam 3 | Q90 | 7 |
| Exam 3 | Q91 | 9 |
| Exam 3 | Q92 | 7 |
| Exam 3 | Q93 | 6 |
| Exam 3 | Q94 | 8 |
| Exam 3 | Q95 | 9 |
| Exam 3 | Q96 | 6 |
| Exam 3 | Q97 | 6 |
| Exam 3 | Q98 | 5 |
| Exam 3 | Q99 | 6 |
| Exam 3 | Q100 | 7 |

Option counts were parsed from the original examination documents. Only scored correctness (0/1), not the options actually selected by residents, was available; distractor-functioning analysis is unsupported. The system form-level difficulty coefficients are separate quantities and were not substituted for item proportions correct.

*Supplementary Table S7. Item proportions correct and discrimination*

p is the proportion correct. r is the corrected correlation with the sum of the other 99 items. Case r is the correlation with the remaining case-subscale items and is shown only for case items. NE indicates zero variance or a non-case item for Case r. The matrices had no missing values. All-record and deduplicated estimates are shown separately. These are exploratory estimates, especially for Exam 1 (n = 27); no item was removed or rescored.

*Exam 1: all-record n = 27; deduplicated n = 27*

| **Item** | **p all** | **r all** | **Case r all** | **p dedup** | **r dedup** | **Case r dedup** |
| --- | --- | --- | --- | --- | --- | --- |
| Q1 | 0.889 | -0.009 | NE | 0.889 | -0.009 | NE |
| Q2 | 0.963 | 0.386 | NE | 0.963 | 0.386 | NE |
| Q3 | 0.889 | -0.163 | NE | 0.889 | -0.163 | NE |
| Q4 | 0.889 | 0.101 | NE | 0.889 | 0.101 | NE |
| Q5 | 0.889 | 0.403 | NE | 0.889 | 0.403 | NE |
| Q6 | 0.889 | 0.164 | NE | 0.889 | 0.164 | NE |
| Q7 | 0.963 | 0.254 | NE | 0.963 | 0.254 | NE |
| Q8 | 0.889 | -0.071 | NE | 0.889 | -0.071 | NE |
| Q9 | 0.889 | 0.007 | NE | 0.889 | 0.007 | NE |
| Q10 | 0.889 | 0.148 | NE | 0.889 | 0.148 | NE |
| Q11 | 0.889 | -0.086 | NE | 0.889 | -0.086 | NE |
| Q12 | 0.889 | 0.132 | NE | 0.889 | 0.132 | NE |
| Q13 | 0.889 | 0.085 | NE | 0.889 | 0.085 | NE |
| Q14 | 0.889 | 0.101 | NE | 0.889 | 0.101 | NE |
| Q15 | 0.889 | -0.055 | NE | 0.889 | -0.055 | NE |
| Q16 | 0.889 | 0.164 | NE | 0.889 | 0.164 | NE |
| Q17 | 0.889 | 0.132 | NE | 0.889 | 0.132 | NE |
| Q18 | 0.889 | 0.101 | NE | 0.889 | 0.101 | NE |
| Q19 | 0.704 | -0.138 | NE | 0.704 | -0.138 | NE |
| Q20 | 0.852 | 0.061 | NE | 0.852 | 0.061 | NE |
| Q21 | 0.852 | 0.033 | NE | 0.852 | 0.033 | NE |
| Q22 | 0.852 | 0.243 | NE | 0.852 | 0.243 | NE |
| Q23 | 0.852 | 0.006 | NE | 0.852 | 0.006 | NE |
| Q24 | 0.852 | 0.500 | NE | 0.852 | 0.500 | NE |
| Q25 | 0.852 | -0.321 | NE | 0.852 | -0.321 | NE |
| Q26 | 0.852 | 0.342 | NE | 0.852 | 0.342 | NE |
| Q27 | 0.852 | -0.049 | NE | 0.852 | -0.049 | NE |
| Q28 | 0.852 | 0.243 | NE | 0.852 | 0.243 | NE |
| Q29 | 0.852 | -0.008 | NE | 0.852 | -0.008 | NE |
| Q30 | 0.852 | 0.075 | NE | 0.852 | 0.075 | NE |
| Q31 | 0.852 | 0.442 | NE | 0.852 | 0.442 | NE |
| Q32 | 0.852 | 0.457 | NE | 0.852 | 0.457 | NE |
| Q33 | 0.852 | -0.186 | NE | 0.852 | -0.186 | NE |
| Q34 | 0.852 | -0.036 | NE | 0.852 | -0.036 | NE |
| Q35 | 0.852 | 0.356 | NE | 0.852 | 0.356 | NE |
| Q36 | 0.852 | 0.075 | NE | 0.852 | 0.075 | NE |
| Q37 | 0.852 | 0.173 | NE | 0.852 | 0.173 | NE |
| Q38 | 0.852 | -0.213 | NE | 0.852 | -0.213 | NE |
| Q39 | 0.852 | 0.371 | NE | 0.852 | 0.371 | NE |
| Q40 | 0.852 | 0.131 | NE | 0.852 | 0.131 | NE |
| Q41 | 0.852 | 0.515 | NE | 0.852 | 0.515 | NE |
| Q42 | 0.852 | 0.457 | NE | 0.852 | 0.457 | NE |
| Q43 | 0.852 | -0.132 | NE | 0.852 | -0.132 | NE |
| Q44 | 0.852 | 0.271 | NE | 0.852 | 0.271 | NE |
| Q45 | 0.852 | -0.077 | NE | 0.852 | -0.077 | NE |
| Q46 | 0.852 | 0.145 | NE | 0.852 | 0.145 | NE |
| Q47 | 0.852 | 0.061 | NE | 0.852 | 0.061 | NE |
| Q48 | 0.852 | 0.019 | NE | 0.852 | 0.019 | NE |
| Q49 | 0.815 | 0.082 | NE | 0.815 | 0.082 | NE |
| Q50 | 0.815 | 0.171 | NE | 0.815 | 0.171 | NE |
| Q51 | 0.815 | 0.197 | NE | 0.815 | 0.197 | NE |
| Q52 | 0.815 | 0.031 | NE | 0.815 | 0.031 | NE |
| Q53 | 0.815 | 0.405 | NE | 0.815 | 0.405 | NE |
| Q54 | 0.815 | 0.018 | NE | 0.815 | 0.018 | NE |
| Q55 | 0.815 | 0.197 | NE | 0.815 | 0.197 | NE |
| Q56 | 0.815 | 0.006 | NE | 0.815 | 0.006 | NE |
| Q57 | 0.815 | 0.418 | NE | 0.815 | 0.418 | NE |
| Q58 | 0.815 | 0.287 | NE | 0.815 | 0.287 | NE |
| Q59 | 0.815 | 0.184 | NE | 0.815 | 0.184 | NE |
| Q60 | 0.815 | 0.261 | NE | 0.815 | 0.261 | NE |
| Q61 | 0.889 | -0.240 | NE | 0.889 | -0.240 | NE |
| Q62 | 0.889 | 0.180 | NE | 0.889 | 0.180 | NE |
| Q63 | 0.889 | 0.038 | NE | 0.889 | 0.038 | NE |
| Q64 | 0.889 | 0.054 | NE | 0.889 | 0.054 | NE |
| Q65 | 0.889 | 0.117 | NE | 0.889 | 0.117 | NE |
| Q66 | 0.889 | 0.085 | NE | 0.889 | 0.085 | NE |
| Q67 | 0.889 | 0.132 | NE | 0.889 | 0.132 | NE |
| Q68 | 0.889 | -0.117 | NE | 0.889 | -0.117 | NE |
| Q69 | 0.889 | 0.085 | NE | 0.889 | 0.085 | NE |
| Q70 | 0.889 | 0.259 | NE | 0.889 | 0.259 | NE |
| Q71 | 0.889 | 0.533 | NE | 0.889 | 0.533 | NE |
| Q72 | 0.889 | 0.211 | NE | 0.889 | 0.211 | NE |
| Q73 | 0.889 | -0.148 | NE | 0.889 | -0.148 | NE |
| Q74 | 0.889 | 0.227 | NE | 0.889 | 0.227 | NE |
| Q75 | 0.852 | 0.033 | NE | 0.852 | 0.033 | NE |
| Q76 | 0.852 | 0.271 | NE | 0.852 | 0.271 | NE |
| Q77 | 0.852 | 0.299 | NE | 0.852 | 0.299 | NE |
| Q78 | 0.852 | 0.500 | NE | 0.852 | 0.500 | NE |
| Q79 | 0.852 | 0.159 | NE | 0.852 | 0.159 | NE |
| Q80 | 0.852 | 0.602 | NE | 0.852 | 0.602 | NE |
| Q81 | 0.852 | 0.631 | NE | 0.852 | 0.631 | NE |
| Q82 | 0.852 | 0.243 | NE | 0.852 | 0.243 | NE |
| Q83 | 0.852 | 0.328 | NE | 0.852 | 0.328 | NE |
| Q84 | 0.852 | 0.019 | NE | 0.852 | 0.019 | NE |
| Q85 | 0.852 | -0.063 | NE | 0.852 | -0.063 | NE |
| Q86 | 0.852 | -0.091 | NE | 0.852 | -0.091 | NE |
| Q87 | 0.852 | 0.117 | NE | 0.852 | 0.117 | NE |
| Q88 | 0.852 | 0.285 | NE | 0.852 | 0.285 | NE |
| Q89 | 0.852 | 0.075 | NE | 0.852 | 0.075 | NE |
| Q90 | 0.593 | 0.487 | 0.372 | 0.593 | 0.487 | 0.372 |
| Q91 | 0.556 | 0.341 | 0.263 | 0.556 | 0.341 | 0.263 |
| Q92 | 0.556 | 0.008 | 0.098 | 0.556 | 0.008 | 0.098 |
| Q93 | 0.556 | 0.018 | 0.130 | 0.556 | 0.018 | 0.130 |
| Q94 | 0.556 | 0.290 | 0.263 | 0.556 | 0.290 | 0.263 |
| Q95 | 0.556 | 0.117 | 0.066 | 0.556 | 0.117 | 0.066 |
| Q96 | 0.556 | 0.341 | 0.333 | 0.556 | 0.341 | 0.333 |
| Q97 | 0.556 | 0.445 | 0.263 | 0.556 | 0.445 | 0.263 |
| Q98 | 0.556 | 0.414 | 0.516 | 0.556 | 0.414 | 0.516 |
| Q99 | 0.556 | 0.445 | 0.404 | 0.556 | 0.445 | 0.404 |
| Q100 | 0.556 | 0.279 | 0.333 | 0.556 | 0.279 | 0.333 |

*Exam 2: all-record n = 235; deduplicated n = 227*

| **Item** | **p all** | **r all** | **Case r all** | **p dedup** | **r dedup** | **Case r dedup** |
| --- | --- | --- | --- | --- | --- | --- |
| Q1 | 0.770 | 0.140 | NE | 0.762 | 0.139 | NE |
| Q2 | 0.770 | 0.135 | NE | 0.767 | 0.137 | NE |
| Q3 | 0.770 | 0.060 | NE | 0.762 | 0.057 | NE |
| Q4 | 0.766 | 0.097 | NE | 0.767 | 0.094 | NE |
| Q5 | 0.766 | 0.095 | NE | 0.767 | 0.084 | NE |
| Q6 | 0.766 | 0.095 | NE | 0.767 | 0.092 | NE |
| Q7 | 0.766 | 0.184 | NE | 0.771 | 0.188 | NE |
| Q8 | 0.766 | 0.131 | NE | 0.771 | 0.161 | NE |
| Q9 | 0.766 | 0.064 | NE | 0.771 | 0.092 | NE |
| Q10 | 0.766 | 0.144 | NE | 0.762 | 0.155 | NE |
| Q11 | 0.766 | 0.174 | NE | 0.762 | 0.176 | NE |
| Q12 | 0.766 | 0.143 | NE | 0.767 | 0.148 | NE |
| Q13 | 0.766 | 0.133 | NE | 0.758 | 0.132 | NE |
| Q14 | 0.766 | 0.082 | NE | 0.762 | 0.076 | NE |
| Q15 | 0.766 | 0.201 | NE | 0.767 | 0.189 | NE |
| Q16 | 0.766 | 0.077 | NE | 0.771 | 0.070 | NE |
| Q17 | 0.766 | -0.022 | NE | 0.762 | -0.029 | NE |
| Q18 | 0.766 | 0.200 | NE | 0.771 | 0.191 | NE |
| Q19 | 0.766 | 0.210 | NE | 0.767 | 0.202 | NE |
| Q20 | 0.766 | 0.112 | NE | 0.767 | 0.097 | NE |
| Q21 | 0.864 | 0.208 | NE | 0.863 | 0.215 | NE |
| Q22 | 0.864 | 0.138 | NE | 0.863 | 0.136 | NE |
| Q23 | 0.864 | 0.089 | NE | 0.868 | 0.089 | NE |
| Q24 | 0.864 | 0.225 | NE | 0.863 | 0.215 | NE |
| Q25 | 0.864 | 0.182 | NE | 0.859 | 0.182 | NE |
| Q26 | 0.864 | 0.225 | NE | 0.859 | 0.226 | NE |
| Q27 | 0.864 | 0.223 | NE | 0.868 | 0.218 | NE |
| Q28 | 0.864 | 0.178 | NE | 0.868 | 0.165 | NE |
| Q29 | 0.864 | 0.183 | NE | 0.859 | 0.184 | NE |
| Q30 | 0.864 | 0.185 | NE | 0.859 | 0.185 | NE |
| Q31 | 0.864 | 0.192 | NE | 0.859 | 0.192 | NE |
| Q32 | 0.864 | 0.020 | NE | 0.863 | 0.029 | NE |
| Q33 | 0.864 | 0.168 | NE | 0.863 | 0.182 | NE |
| Q34 | 0.864 | 0.190 | NE | 0.872 | 0.191 | NE |
| Q35 | 0.864 | 0.197 | NE | 0.872 | 0.200 | NE |
| Q36 | 0.864 | 0.168 | NE | 0.868 | 0.174 | NE |
| Q37 | 0.864 | 0.141 | NE | 0.863 | 0.137 | NE |
| Q38 | 0.864 | 0.211 | NE | 0.859 | 0.212 | NE |
| Q39 | 0.864 | 0.234 | NE | 0.863 | 0.233 | NE |
| Q40 | 0.864 | 0.185 | NE | 0.872 | 0.187 | NE |
| Q41 | 0.864 | 0.156 | NE | 0.863 | 0.172 | NE |
| Q42 | 0.864 | 0.196 | NE | 0.868 | 0.192 | NE |
| Q43 | 0.864 | 0.171 | NE | 0.868 | 0.187 | NE |
| Q44 | 0.860 | 0.147 | NE | 0.859 | 0.163 | NE |
| Q45 | 0.860 | 0.154 | NE | 0.855 | 0.153 | NE |
| Q46 | 0.860 | 0.238 | NE | 0.859 | 0.238 | NE |
| Q47 | 0.860 | 0.279 | NE | 0.855 | 0.281 | NE |
| Q48 | 0.860 | 0.146 | NE | 0.855 | 0.145 | NE |
| Q49 | 0.860 | 0.090 | NE | 0.855 | 0.089 | NE |
| Q50 | 0.860 | 0.237 | NE | 0.863 | 0.270 | NE |
| Q51 | 0.868 | 0.193 | NE | 0.868 | 0.182 | NE |
| Q52 | 0.868 | 0.188 | NE | 0.868 | 0.194 | NE |
| Q53 | 0.868 | 0.241 | NE | 0.863 | 0.242 | NE |
| Q54 | 0.868 | 0.125 | NE | 0.868 | 0.129 | NE |
| Q55 | 0.868 | 0.119 | NE | 0.872 | 0.149 | NE |
| Q56 | 0.868 | -0.043 | NE | 0.863 | -0.047 | NE |
| Q57 | 0.868 | 0.179 | NE | 0.872 | 0.165 | NE |
| Q58 | 0.868 | 0.064 | NE | 0.877 | 0.085 | NE |
| Q59 | 0.868 | 0.161 | NE | 0.863 | 0.161 | NE |
| Q60 | 0.868 | 0.097 | NE | 0.863 | 0.096 | NE |
| Q61 | 0.868 | 0.161 | NE | 0.872 | 0.147 | NE |
| Q62 | 0.868 | 0.117 | NE | 0.863 | 0.116 | NE |
| Q63 | 0.868 | 0.249 | NE | 0.868 | 0.240 | NE |
| Q64 | 0.868 | 0.135 | NE | 0.868 | 0.131 | NE |
| Q65 | 0.868 | 0.088 | NE | 0.863 | 0.086 | NE |
| Q66 | 0.868 | 0.028 | NE | 0.877 | 0.021 | NE |
| Q67 | 0.868 | 0.077 | NE | 0.868 | 0.072 | NE |
| Q68 | 0.868 | 0.089 | NE | 0.872 | 0.117 | NE |
| Q69 | 0.868 | 0.234 | NE | 0.868 | 0.233 | NE |
| Q70 | 0.868 | 0.199 | NE | 0.868 | 0.197 | NE |
| Q71 | 0.868 | 0.119 | NE | 0.868 | 0.132 | NE |
| Q72 | 0.868 | 0.133 | NE | 0.872 | 0.147 | NE |
| Q73 | 0.868 | 0.215 | NE | 0.868 | 0.214 | NE |
| Q74 | 0.868 | 0.179 | NE | 0.872 | 0.172 | NE |
| Q75 | 0.868 | 0.204 | NE | 0.872 | 0.216 | NE |
| Q76 | 0.868 | 0.188 | NE | 0.863 | 0.188 | NE |
| Q77 | 0.868 | 0.100 | NE | 0.863 | 0.099 | NE |
| Q78 | 0.868 | 0.131 | NE | 0.868 | 0.128 | NE |
| Q79 | 0.868 | 0.149 | NE | 0.863 | 0.148 | NE |
| Q80 | 0.864 | 0.144 | NE | 0.859 | 0.143 | NE |
| Q81 | 0.864 | 0.145 | NE | 0.863 | 0.133 | NE |
| Q82 | 0.604 | 0.341 | 0.383 | 0.608 | 0.351 | 0.386 |
| Q83 | 0.604 | 0.326 | 0.331 | 0.608 | 0.339 | 0.343 |
| Q84 | 0.604 | 0.414 | 0.400 | 0.599 | 0.413 | 0.407 |
| Q85 | 0.600 | 0.358 | 0.398 | 0.590 | 0.352 | 0.391 |
| Q86 | 0.600 | 0.351 | 0.444 | 0.586 | 0.355 | 0.449 |
| Q87 | 0.600 | 0.376 | 0.433 | 0.604 | 0.377 | 0.435 |
| Q88 | 0.600 | 0.361 | 0.400 | 0.599 | 0.353 | 0.387 |
| Q89 | 0.600 | 0.286 | 0.348 | 0.595 | 0.295 | 0.353 |
| Q90 | 0.600 | 0.393 | 0.452 | 0.599 | 0.393 | 0.450 |
| Q91 | 0.600 | 0.352 | 0.380 | 0.599 | 0.357 | 0.396 |
| Q92 | 0.600 | 0.256 | 0.275 | 0.599 | 0.247 | 0.276 |
| Q93 | 0.600 | 0.302 | 0.305 | 0.595 | 0.309 | 0.314 |
| Q94 | 0.600 | 0.248 | 0.307 | 0.599 | 0.237 | 0.292 |
| Q95 | 0.600 | 0.359 | 0.337 | 0.604 | 0.361 | 0.336 |
| Q96 | 0.600 | 0.390 | 0.398 | 0.595 | 0.382 | 0.385 |
| Q97 | 0.600 | 0.422 | 0.437 | 0.595 | 0.420 | 0.432 |
| Q98 | 0.600 | 0.448 | 0.457 | 0.599 | 0.444 | 0.448 |
| Q99 | 0.600 | 0.296 | 0.391 | 0.599 | 0.282 | 0.387 |
| Q100 | 0.600 | 0.300 | 0.335 | 0.590 | 0.305 | 0.340 |

*Exam 3: all-record n = 175; deduplicated n = 158*

| **Item** | **p all** | **r all** | **Case r all** | **p dedup** | **r dedup** | **Case r dedup** |
| --- | --- | --- | --- | --- | --- | --- |
| Q1 | 0.989 | 0.152 | NE | 0.987 | 0.183 | NE |
| Q2 | 1.000 | NE | NE | 1.000 | NE | NE |
| Q3 | 0.960 | 0.292 | NE | 0.956 | 0.355 | NE |
| Q4 | 0.731 | 0.436 | NE | 0.759 | 0.388 | NE |
| Q5 | 1.000 | NE | NE | 1.000 | NE | NE |
| Q6 | 0.966 | 0.260 | NE | 0.962 | 0.315 | NE |
| Q7 | 1.000 | NE | NE | 1.000 | NE | NE |
| Q8 | 1.000 | NE | NE | 1.000 | NE | NE |
| Q9 | 1.000 | NE | NE | 1.000 | NE | NE |
| Q10 | 1.000 | NE | NE | 1.000 | NE | NE |
| Q11 | 0.994 | 0.196 | NE | 0.994 | 0.232 | NE |
| Q12 | 1.000 | NE | NE | 1.000 | NE | NE |
| Q13 | 1.000 | NE | NE | 1.000 | NE | NE |
| Q14 | 0.857 | 0.400 | NE | 0.873 | 0.383 | NE |
| Q15 | 0.474 | 0.414 | NE | 0.494 | 0.403 | NE |
| Q16 | 0.126 | 0.277 | NE | 0.139 | 0.295 | NE |
| Q17 | 0.931 | 0.323 | NE | 0.930 | 0.346 | NE |
| Q18 | 1.000 | NE | NE | 1.000 | NE | NE |
| Q19 | 0.914 | 0.390 | NE | 0.930 | 0.346 | NE |
| Q20 | 0.960 | 0.292 | NE | 0.956 | 0.355 | NE |
| Q21 | 0.891 | 0.171 | NE | 0.892 | 0.232 | NE |
| Q22 | 0.891 | 0.077 | NE | 0.905 | -0.001 | NE |
| Q23 | 0.891 | 0.102 | NE | 0.899 | 0.086 | NE |
| Q24 | 0.891 | 0.212 | NE | 0.911 | 0.167 | NE |
| Q25 | 0.891 | 0.134 | NE | 0.905 | 0.034 | NE |
| Q26 | 0.891 | 0.129 | NE | 0.899 | 0.032 | NE |
| Q27 | 0.891 | 0.036 | NE | 0.886 | 0.052 | NE |
| Q28 | 0.891 | 0.136 | NE | 0.905 | 0.107 | NE |
| Q29 | 0.891 | 0.086 | NE | 0.892 | 0.049 | NE |
| Q30 | 0.891 | 0.002 | NE | 0.899 | -0.105 | NE |
| Q31 | 0.891 | 0.229 | NE | 0.905 | 0.045 | NE |
| Q32 | 0.891 | -0.078 | NE | 0.880 | -0.063 | NE |
| Q33 | 0.891 | 0.068 | NE | 0.886 | 0.090 | NE |
| Q34 | 0.886 | -0.003 | NE | 0.899 | 0.017 | NE |
| Q35 | 0.886 | 0.075 | NE | 0.892 | 0.136 | NE |
| Q36 | 0.886 | 0.026 | NE | 0.880 | -0.026 | NE |
| Q37 | 0.886 | 0.120 | NE | 0.886 | 0.181 | NE |
| Q38 | 0.886 | 0.064 | NE | 0.886 | 0.121 | NE |
| Q39 | 0.886 | 0.044 | NE | 0.880 | 0.087 | NE |
| Q40 | 0.886 | 0.120 | NE | 0.892 | 0.016 | NE |
| Q41 | 0.880 | 0.279 | NE | 0.892 | 0.209 | NE |
| Q42 | 0.977 | 0.283 | NE | 0.981 | 0.185 | NE |
| Q43 | 0.897 | 0.300 | NE | 0.899 | 0.274 | NE |
| Q44 | 0.920 | 0.464 | NE | 0.930 | 0.377 | NE |
| Q45 | 0.891 | 0.194 | NE | 0.905 | 0.146 | NE |
| Q46 | 0.971 | 0.194 | NE | 0.981 | 0.071 | NE |
| Q47 | 0.886 | 0.271 | NE | 0.886 | 0.250 | NE |
| Q48 | 0.909 | 0.229 | NE | 0.905 | 0.202 | NE |
| Q49 | 0.909 | 0.157 | NE | 0.918 | 0.064 | NE |
| Q50 | 0.903 | 0.232 | NE | 0.918 | 0.263 | NE |
| Q51 | 0.903 | 0.325 | NE | 0.918 | 0.196 | NE |
| Q52 | 0.880 | 0.175 | NE | 0.886 | 0.145 | NE |
| Q53 | 0.880 | 0.306 | NE | 0.899 | 0.204 | NE |
| Q54 | 0.977 | 0.221 | NE | 0.981 | 0.178 | NE |
| Q55 | 0.880 | 0.188 | NE | 0.873 | 0.202 | NE |
| Q56 | 0.989 | 0.353 | NE | 0.994 | 0.232 | NE |
| Q57 | 0.903 | 0.150 | NE | 0.899 | 0.143 | NE |
| Q58 | 0.960 | 0.288 | NE | 0.968 | 0.228 | NE |
| Q59 | 1.000 | NE | NE | 1.000 | NE | NE |
| Q60 | 0.909 | 0.249 | NE | 0.911 | 0.241 | NE |
| Q61 | 0.891 | 0.201 | NE | 0.886 | 0.178 | NE |
| Q62 | 0.874 | 0.118 | NE | 0.886 | 0.042 | NE |
| Q63 | 0.874 | 0.181 | NE | 0.886 | 0.099 | NE |
| Q64 | 0.960 | 0.307 | NE | 0.975 | 0.191 | NE |
| Q65 | 0.880 | 0.160 | NE | 0.880 | 0.216 | NE |
| Q66 | 0.880 | 0.050 | NE | 0.873 | 0.087 | NE |
| Q67 | 0.869 | 0.243 | NE | 0.867 | 0.249 | NE |
| Q68 | 0.869 | 0.139 | NE | 0.886 | 0.071 | NE |
| Q69 | 0.869 | 0.263 | NE | 0.880 | 0.202 | NE |
| Q70 | 0.874 | 0.137 | NE | 0.873 | 0.129 | NE |
| Q71 | 0.874 | 0.139 | NE | 0.880 | 0.077 | NE |
| Q72 | 0.869 | 0.163 | NE | 0.867 | 0.090 | NE |
| Q73 | 0.623 | 0.320 | NE | 0.627 | 0.297 | NE |
| Q74 | 0.869 | 0.256 | NE | 0.861 | 0.254 | NE |
| Q75 | 0.869 | 0.218 | NE | 0.892 | 0.058 | NE |
| Q76 | 0.869 | 0.199 | NE | 0.867 | 0.205 | NE |
| Q77 | 0.029 | 0.199 | NE | 0.032 | 0.218 | NE |
| Q78 | 0.863 | 0.164 | NE | 0.854 | 0.146 | NE |
| Q79 | 0.874 | 0.163 | NE | 0.873 | 0.166 | NE |
| Q80 | 0.874 | 0.209 | NE | 0.886 | 0.129 | NE |
| Q81 | 0.880 | 0.052 | NE | 0.886 | 0.036 | NE |
| Q82 | 0.554 | 0.396 | 0.497 | 0.570 | 0.388 | 0.499 |
| Q83 | 0.549 | 0.360 | 0.314 | 0.563 | 0.306 | 0.255 |
| Q84 | 0.549 | 0.340 | 0.342 | 0.557 | 0.314 | 0.292 |
| Q85 | 0.549 | 0.280 | 0.335 | 0.551 | 0.296 | 0.297 |
| Q86 | 0.549 | 0.288 | 0.314 | 0.563 | 0.285 | 0.280 |
| Q87 | 0.549 | 0.250 | 0.268 | 0.570 | 0.170 | 0.204 |
| Q88 | 0.549 | 0.267 | 0.249 | 0.551 | 0.244 | 0.222 |
| Q89 | 0.549 | 0.359 | 0.280 | 0.563 | 0.364 | 0.255 |
| Q90 | 0.549 | 0.368 | 0.530 | 0.551 | 0.388 | 0.502 |
| Q91 | 0.549 | 0.185 | 0.308 | 0.557 | 0.194 | 0.299 |
| Q92 | 0.549 | 0.251 | 0.258 | 0.551 | 0.215 | 0.226 |
| Q93 | 0.549 | 0.387 | 0.481 | 0.570 | 0.361 | 0.450 |
| Q94 | 0.549 | 0.297 | 0.320 | 0.551 | 0.247 | 0.279 |
| Q95 | 0.549 | 0.223 | 0.201 | 0.532 | 0.289 | 0.241 |
| Q96 | 0.549 | 0.187 | 0.261 | 0.563 | 0.167 | 0.269 |
| Q97 | 0.549 | 0.212 | 0.289 | 0.557 | 0.226 | 0.295 |
| Q98 | 0.549 | 0.313 | 0.308 | 0.544 | 0.323 | 0.312 |
| Q99 | 0.549 | 0.236 | 0.225 | 0.563 | 0.177 | 0.167 |
| Q100 | 0.549 | 0.206 | 0.314 | 0.557 | 0.236 | 0.302 |

*Supplementary Table S8. Full-form and case-subscale reliability*

Alpha equals KR-20 for these complete binary scores. Each form is calculated separately; no pooled cross-form alpha is reported. Percentile 95% bootstrap CIs use 2,000 samples and seed 20260907, restarted for each form/cohort. Residents are resampled as clusters; within-form repeat records stay together. All 2,000 resamples yielded finite estimates in each analysis. These intervals quantify sampling uncertainty, not validity or equivalence.

All records within each examination form

| **Form** | **Scale** | **Records** | **Residents** | **Items** | **Alpha** | **95% CI** |
| --- | --- | --- | --- | --- | --- | --- |
| Exam 1 | full form | 27 | 27 | 100 | 0.775 | 0.520–0.863 |
| Exam 1 | case subscale | 27 | 27 | 11 | 0.613 | 0.430–0.724 |
| Exam 2 | full form | 235 | 234 | 100 | 0.827 | 0.797–0.850 |
| Exam 2 | case subscale | 235 | 234 | 19 | 0.807 | 0.767–0.838 |
| Exam 3 | full form | 175 | 174 | 100 | 0.835 | 0.784–0.870 |
| Exam 3 | case subscale | 175 | 174 | 19 | 0.752 | 0.694–0.798 |

*One retained record per resident*

| **Form** | **Scale** | **Records** | **Residents** | **Items** | **Alpha** | **95% CI** |
| --- | --- | --- | --- | --- | --- | --- |
| Exam 1 | full form | 27 | 27 | 100 | 0.775 | 0.520–0.863 |
| Exam 1 | case subscale | 27 | 27 | 11 | 0.613 | 0.430–0.724 |
| Exam 2 | full form | 227 | 227 | 100 | 0.828 | 0.799–0.851 |
| Exam 2 | case subscale | 227 | 227 | 19 | 0.807 | 0.767–0.839 |
| Exam 3 | full form | 158 | 158 | 100 | 0.805 | 0.753–0.844 |
| Exam 3 | case subscale | 158 | 158 | 19 | 0.726 | 0.658–0.774 |

*Supplementary Table S9. Exploratory Exam 2 DIF screening*

The five-specialty sample included 221 residents: internal medicine 54, surgery 77, anesthesiology 20, orthopedics 21, and other clinical specialties 49. Critical care n = 6 was excluded from DIF estimation. The matching variable was the standardized sum of the other 99 items. M0 used the matching score; M1 added specialty; M2 added specialty-by-matching-score interactions. The omnibus test compared M2 with M0; uniform and nonuniform tests compared M1/M0 and M2/M1. Internal medicine was the reference category. ΔR² denotes the increment in Nagelkerke R² from M0 to M2.

A minimum of five correct and five incorrect responses in every specialty was required. Twenty-one items were estimable; 79 failed this minimum-cell rule. No additional model warning excluded an otherwise eligible item. Benjamini–Hochberg correction preserved a family size of 100 for each test type using internal p=1 placeholders for non-estimable items; those items are not reported as having valid p values. No omnibus, uniform, or nonuniform q was below .05. This does not establish absence of DIF or measurement invariance.

| **Item** | **Omnibus p** | **q** | **Uniform p** | **q** | **Nonuniform p** | **q** | **ΔR²** |
| --- | --- | --- | --- | --- | --- | --- | --- |
| Q1 | 0.961 | 1.000 | 0.957 | 1.000 | 0.761 | 1.000 | 0.017 |
| Q2 | 0.015 | 1.000 | 0.370 | 1.000 | 0.006 | 0.521 | 0.121 |
| Q14 | 0.288 | 1.000 | 0.259 | 1.000 | 0.355 | 1.000 | 0.064 |
| Q19 | 0.673 | 1.000 | 0.577 | 1.000 | 0.579 | 1.000 | 0.037 |
| Q82 | 0.042 | 1.000 | 0.580 | 1.000 | 0.010 | 0.521 | 0.083 |
| Q83 | 0.720 | 1.000 | 0.613 | 1.000 | 0.615 | 1.000 | 0.029 |
| Q84 | 0.079 | 1.000 | 0.053 | 1.000 | 0.312 | 1.000 | 0.070 |
| Q85 | 0.318 | 1.000 | 0.839 | 1.000 | 0.097 | 1.000 | 0.048 |
| Q86 | 0.206 | 1.000 | 0.105 | 1.000 | 0.515 | 1.000 | 0.057 |
| Q87 | 0.625 | 1.000 | 0.621 | 1.000 | 0.468 | 1.000 | 0.032 |
| Q89 | 0.381 | 1.000 | 0.342 | 1.000 | 0.398 | 1.000 | 0.047 |
| Q90 | 0.646 | 1.000 | 0.265 | 1.000 | 0.941 | 1.000 | 0.031 |
| Q91 | 0.289 | 1.000 | 0.494 | 1.000 | 0.179 | 1.000 | 0.050 |
| Q92 | 0.526 | 1.000 | 0.410 | 1.000 | 0.536 | 1.000 | 0.040 |
| Q93 | 0.453 | 1.000 | 0.198 | 1.000 | 0.773 | 1.000 | 0.043 |
| Q94 | 0.225 | 1.000 | 0.428 | 1.000 | 0.149 | 1.000 | 0.059 |
| Q95 | 0.415 | 1.000 | 0.161 | 1.000 | 0.803 | 1.000 | 0.043 |
| Q96 | 0.123 | 1.000 | 0.545 | 1.000 | 0.048 | 1.000 | 0.065 |
| Q98 | 0.634 | 1.000 | 0.598 | 1.000 | 0.500 | 1.000 | 0.029 |
| Q99 | 0.459 | 1.000 | 0.703 | 1.000 | 0.234 | 1.000 | 0.042 |
| Q100 | 0.599 | 1.000 | 0.717 | 1.000 | 0.363 | 1.000 | 0.035 |

Not stably estimable (sparse group response cells): Q3, Q4, Q5, Q6, Q7, Q8, Q9, Q10, Q11, Q12, Q13, Q15, Q16, Q17, Q18, Q20, Q21, Q22, Q23, Q24, Q25, Q26, Q27, Q28, Q29, Q30, Q31, Q32, Q33, Q34, Q35, Q36, Q37, Q38, Q39, Q40, Q41, Q42, Q43, Q44, Q45, Q46, Q47, Q48, Q49, Q50, Q51, Q52, Q53, Q54, Q55, Q56, Q57, Q58, Q59, Q60, Q61, Q62, Q63, Q64, Q65, Q66, Q67, Q68, Q69, Q70, Q71, Q72, Q73, Q74, Q75, Q76, Q77, Q78, Q79, Q80, Q81, Q88, Q97.

The matching score was not established as an invariant anchor; limited sample size, sparse groups, matching-score specification, and multiple testing constrain sensitivity. Results apply only to the estimable subset. No stable item-level DIF inference was attempted for Exam 1 or for the six critical-care residents. Complete model coefficients and item-level diagnostics are retained in the revision analysis archive.

**Supplementary Table S10. Sensitivity to alternative specialty classifications**

All analyses use the same locked individual total scores as the original analyses. Original denotes the six-category coding used in Tables 1–3 and S2–S5. A merges Orthopedics and the one separately coded Plastic surgery record into Surgery. B additionally moves Neurology and Infectious diseases from Other Clinical to Internal Medicine. C additionally assigns the one PGY-3 record originally carrying a non-specialty status label to Anesthesiology, as indicated by its source department and specialty fields. These are alternative analytical definitions, not a claim that one universal specialty taxonomy applies.

| **Cohort** | **Coding** | **n** | **F** | **df** | **p** | **η²** |
| --- | --- | --- | --- | --- | --- | --- |
| 412 residents | Original | 412 | 8.655 | 5, 406 | <0.001 | 0.0963 |
| 412 residents | A | 412 | 8.044 | 4, 407 | <0.001 | 0.0733 |
| 412 residents | B | 412 | 8.006 | 4, 407 | <0.001 | 0.0729 |
| 412 residents | C | 412 | 7.926 | 4, 407 | <0.001 | 0.0723 |
| PGY-2 | Original | 227 | 4.814 | 5, 221 | <0.001 | 0.0982 |
| PGY-2 | A | 227 | 3.166 | 4, 222 | 0.0148 | 0.0540 |
| PGY-2 | B | 227 | 3.265 | 4, 222 | 0.0126 | 0.0556 |

Overall ANOVA tests do not establish significance for any particular pair of specialties. Scheme C is identical to B in PGY-2 because the additional record is PGY-3. The plastic-surgery and infectious-diseases records newly reassigned in A/B are also outside PGY-2. The four residents already coded as surgery with a plastic-surgery direction remain in Surgery throughout. No individual score was changed using the auxiliary export.

**PGY-2 total-score descriptives**

| **Coding** | **Group** | **n** | **Mean** | **SD** | **95% CI** |
| --- | --- | --- | --- | --- | --- |
| A | Anesthesiology | 20 | 78.10 | 7.62 | 74.53–81.67 |
| A | Critical Care | 6 | 76.83 | 12.70 | 63.50–90.16 |
| A | Internal Medicine | 54 | 82.04 | 9.30 | 79.50–84.57 |
| A | Other Clinical | 49 | 81.51 | 8.40 | 79.10–83.92 |
| A | Surgery | 98 | 77.46 | 9.23 | 75.61–79.31 |
| B | Anesthesiology | 20 | 78.10 | 7.62 | 74.53–81.67 |
| B | Critical Care | 6 | 76.83 | 12.70 | 63.50–90.16 |
| B | Internal Medicine | 70 | 81.37 | 9.27 | 79.16–83.58 |
| B | Other Clinical | 33 | 82.67 | 7.92 | 79.86–85.47 |
| B | Surgery | 98 | 77.46 | 9.23 | 75.61–79.31 |

**All-resident total-score descriptives**

| **Coding** | **Group** | **n** | **Mean** | **SD** | **95% CI** |
| --- | --- | --- | --- | --- | --- |
| A | Anesthesiology | 31 | 77.23 | 8.02 | 74.28–80.17 |
| A | Critical Care | 20 | 80.55 | 8.29 | 76.67–84.43 |
| A | Internal Medicine | 156 | 82.98 | 7.99 | 81.72–84.24 |
| A | Other Clinical | 77 | 82.31 | 7.98 | 80.50–84.12 |
| A | Surgery | 128 | 78.15 | 8.89 | 76.59–79.70 |
| B | Anesthesiology | 31 | 77.23 | 8.02 | 74.28–80.17 |
| B | Critical Care | 20 | 80.55 | 8.29 | 76.67–84.43 |
| B | Internal Medicine | 182 | 82.63 | 8.10 | 81.45–83.82 |
| B | Other Clinical | 51 | 83.22 | 7.59 | 81.08–85.35 |
| B | Surgery | 128 | 78.15 | 8.89 | 76.59–79.70 |
| C | Anesthesiology | 32 | 77.38 | 7.94 | 74.51–80.24 |
| C | Critical Care | 20 | 80.55 | 8.29 | 76.67–84.43 |
| C | Internal Medicine | 182 | 82.63 | 8.10 | 81.45–83.82 |
| C | Other Clinical | 50 | 83.24 | 7.67 | 81.06–85.42 |
| C | Surgery | 128 | 78.15 | 8.89 | 76.59–79.70 |

CIs describe raw group means using the t distribution. In PGY-2, the Surgery mean under A/B is 77.46, while Critical Care has the lowest mean (76.83; n = 6). In all residents, Anesthesiology has the lowest mean under A–C. Thus, the magnitude and ranking are classification-sensitive. In PGY-2, the merged Surgery case-analysis mean is 54.89%, compared with the overall mean of 59.77% and the Anesthesiology mean of 52.37%; these descriptive differences do not test learning-transfer distance.

Source fields distinguish department from specialty and are incomplete for some records. The retained record of one resident was originally coded as neurology, whereas later records were coded as anesthesiology; the earlier source specialty is missing. Its historical coding was retained in these analyses rather than inferred from later records. The alternative classifications do not resolve this uncertainty.

**Supplementary Table S11. Distribution of additional records and descriptive comparisons**

The retained dataset contains 412 residents; the full dataset contains 437 records from those residents. The first record in the established dataset was retained for each resident. Twenty-three residents contributed 25 additional records. Counts below follow the specialty coding of each record.

| **Group** | **Retained n** | **All records** | **Additional** | **Retained mean** | **All-record mean** |
| --- | --- | --- | --- | --- | --- |
| Internal Medicine | 156 | 159 | 3 | 82.98 | 82.63 |
| Surgery | 98 | 98 | 0 | 76.86 | 76.86 |
| Anesthesiology | 31 | 46 | 15 | 77.23 | 77.74 |
| Orthopedics | 29 | 29 | 0 | 82.31 | 82.31 |
| Critical Care | 20 | 27 | 7 | 80.55 | 81.33 |
| Other Clinical | 78 | 78 | 0 | 82.33 | 82.33 |

Additional records were concentrated in Anesthesiology and Critical Care. The 23 repeat contributors, classified by their retained record, comprised Internal Medicine 3, Anesthesiology 12, Critical Care 7, and Other Clinical 1. The Other Clinical contributor had two additional records coded as Anesthesiology; consequently, zero additional records coded as Other Clinical does not mean that none of its retained residents had repeat records. This distinction also explains why 15 additional anesthesiology records do not represent 15 residents.

Under the original coding, total-score η² was 0.096 in the deduplicated cohort and 0.082 in the full-record reference (S4); the ordering of the six raw group means was unchanged. The largest absolute group-mean change was 0.78 points in Critical Care. These comparisons show how estimates change when additional records are retained; they do not establish absence of selection bias. Full-record observations are non-independent, and their conventional intervals in S5 are descriptive only. All-record estimates are not an independent replication.

**Additional first-versus-last-record comparison**

| **Group** | **First n** | **Last n** | **First mean** | **Last mean** | **Last − first** |
| --- | --- | --- | --- | --- | --- |
| Internal Medicine | 156 | 156 | 82.98 | 82.58 | -0.40 |
| Surgery | 98 | 98 | 76.86 | 76.86 | +0.00 |
| Anesthesiology | 31 | 32 | 77.23 | 77.41 | +0.18 |
| Orthopedics | 29 | 29 | 82.31 | 82.31 | +0.00 |
| Critical Care | 20 | 20 | 80.55 | 80.30 | -0.25 |
| Other Clinical | 78 | 77 | 82.33 | 82.19 | -0.14 |

First and last refer to record order in the established dataset, not independently verified chronological examination dates. The last-record comparison retained 412 residents and gave F(5,406) = 7.06, η² = 0.080, p < 0.001. The raw-mean ranking was unchanged; the largest first-to-last change was 0.40 points (Internal Medicine). One resident changed from Other Clinical to Anesthesiology under last-record coding, explaining the group-size changes. This descriptive check does not establish absence of selection bias. Of all departmental records, 15/46 (32.6%) in Anesthesiology and 7/27 (25.9%) in Critical Care were additional records; these are record percentages, not percentages of residents.
